# Loss of KCNQ2 or KCNQ3 Leads to Multifocal Time-Varying Activity in the Neonatal Forebrain *Ex Vivo*

**DOI:** 10.1523/ENEURO.0024-21.2021

**Published:** 2021-05-14

**Authors:** Bowen Hou, Nissi Varghese, Heun Soh, Sabato Santaniello, Anastasios V. Tzingounis

**Affiliations:** 1 Department of Physiology and Neurobiology, University of Connecticut, Storrs, CT 06269; 2 Department of Biomedical Engineering, University of Connecticut, Storrs, CT 06269

**Keywords:** channelopathy, epilepsy, KCNQ2, KCNQ3, neurodevelopmental disorders, neurology

## Abstract

Epileptic encephalopathies represent a group of disorders often characterized by refractory seizures, regression in cognitive development, and typically poor prognosis. Dysfunction of KCNQ2 and KCNQ3 channels has emerged as a major cause of neonatal epilepsy. However, our understanding of the cellular mechanisms that may both explain the origins of epilepsy and inform treatment strategies for KCNQ2 and KCNQ3 dysfunction is still lacking. Here, using mesoscale calcium imaging and pharmacology, we demonstrate that in mouse neonatal brain slices, conditional loss of *Kcnq2* from forebrain excitatory neurons (*Pyr:Kcnq2* mice) or constitutive deletion of *Kcnq3* leads to sprawling hyperactivity across the neocortex. Surprisingly, the generation of time-varying hypersynchrony in slices from *Pyr:Kcnq2* mice does not require fast synaptic transmission. This is in contrast to control littermates and constitutive *Kcnq3* knock-out mice where activity is primarily driven by fast synaptic transmission in the neocortex. Unlike in the neocortex, hypersynchronous activity in the hippocampal formation from *Kcnq2* conditional and *Kcnq3* constitutive knock-out mice persists in the presence of synaptic transmission blockers. Thus, we propose that loss of KCNQ2 or KCNQ3 function differentially leads to network hyperactivity across the forebrain in a region-specific and macro-circuit-specific manner.

## Significance Statement

Neocortical hypersynchrony is a hallmark of neonatal epilepsy but its cellular mechanisms are unclear. This study shows that hypersynchrony in the neocortex can stem from the loss of KCNQ2 function in excitatory neurons even in the absence of fast synaptic transmission, unlike the hypersynchrony in response to KCNQ3 loss in the neocortex. This points to unique network dysfunctions involving potassium KCNQ2 channels as a mechanism for neonatal epilepsy.

## Introduction

Ion channelopathies have been associated with multiple neurodevelopmental disorders over the last decades (Satterstrom et al., 2020; Wang et al., 2020). The KCNQ family, otherwise known as the Kv7 family, is a potassium channel family that has been propelled at the forefront of neurodevelopmental disorders (Geisheker et al., 2017; Cornet et al., 2018). Several of its members (KCNQ2-KCNQ5) are expressed in the brain, across multiple brain regions and cell types (Greene and Hoshi, 2017). KCNQ2 channels were the second members of this family to be identified, with a rich history relating to neurodevelopmental disorders. *KCNQ2* was first discovered over 20 years ago as a gene likely to cause benign familial neonatal seizures, a form of a self-limiting pediatric epilepsy disorder (Jentsch, 2000). However, since 2012, multiple studies have shown that *KCNQ2* variants may lead to a large spectrum of symptoms and disorders, including Ohtahara syndrome and autism spectrum disorders (Cornet et al., 2018). These studies have demonstrated that *KCNQ2* pathogenicity leads to developmental and epileptic encephalopathies (DEEs).

Over the years, our knowledge regarding KCNQ2 channel function in the brain has grown exponentially. This understanding has primarily arisen from the use of *Kcnq2* transgenic mice and pharmacological agents that generally target KCNQ2-containing channels. KCNQ2 channels partner with KCNQ3 channels to form heteromeric channels containing two KCNQ2 and two KCNQ3 channels, classically referred to as the M-channels, as they mediate the M-current, a ubiquitous potassium conductance discovered in the 1980s (Jentsch, 2000). KCNQ2/3 channels are strategically located at sites of action potential initiation and regeneration, allowing them to control neuronal excitability by regulating the resting membrane potential of the axon initial segment and axons by preventing excessive firing (Cooper, 2011). Indeed, *Kcnq2* deletion or expression of KCNQ2 pathogenic variants in neurons leads to elevated excitability, typically manifested as a higher number of action potentials and reduced spike frequency adaptation. Such changes in excitability occur in multiple brain regions, including the hippocampus and neocortex, and across multiple cell types (Peters et al., 2005; Singh et al., 2008; Soh et al., 2014, 2018; Verneuil et al., 2020).

To date, most studies on KCNQ2 and KCNQ3 channels using transgenic mice have focused on excitatory neurons in juvenile and adult mice. However, KCNQ2 channels are expressed early in development, with the mRNA expressed before birth (Kanaumi et al., 2008). Indeed, application of the pan-KCNQ blockers XE991 or linopirdine in brain slices from neonatal mice leads to increased excitability, providing evidence that KCNQ channel expression early in life prevents runaway excitation (Okada et al., 2003; Qiu et al., 2007). Additionally, overexpression of dominant-negative KCNQ2 channels in the brain during only the first of week of life results in seizures and premature lethality, suggesting that KCNQ2 channel expression is paramount during the neonatal period (Peters et al., 2005).

Loss-of-function KCNQ2 variants lead to severe neurodevelopmental disorders that present soon after birth. However, the manner in which KCNQ2 dysfunction alters cellular and network excitability early in development is currently unknown. Importantly, it remains unclear how the loss of KCNQ2 channels from excitatory neurons regulates developing hippocampal and neocortical circuits. To capture the dynamics and population activity across the forebrain in the presence and absence of KCNQ2 channels from excitatory neurons, we applied wide-field mesoscale calcium imaging from *Kcnq2* conditional knock-out mice (*Pyr:Kcnq2*) and compared them with constitutive *Kcnq3* knock-out mice. This approach allowed us to image the network dynamics of different forebrain circuits. We found that deletion of *Kcnq2* or *Kcnq3* resulted in multifocal and time-varying excitation across the forebrain. The spontaneous neuronal activity detected in *Pyr:Kcnq2* slices was limited by ongoing GABAA receptor activity and amplified by fast glutamatergic transmission. Importantly, in slices from *Pyr:Kcnq2* mice low-frequency (LF) activity persisted in the absence of fast synaptic transmission in the neocortex, unlike in slices from control or *Kcnq3*-null mice. Thus, ablation of *Kcnq2* channels from excitatory neurons leads to distinct aberrant activity with a unique pharmacological profile.

## Materials and Methods

### Animals

All experiments were performed according to the guidelines described in the National Institutes of Health *Guide for the Care and Use of Laboratory Animals* and were approved by the Institutional Animal Care and Use Committee of the University of Connecticut.

All experimental procedures were performed on mice kept under a 14/10 h light/dark cycle with access to food and water *ad libitum*. *Kcnq2^f/f^* mice on a C57BL/6J background were previously developed and described by our group and others (Soh et al., 2014; Carver and Shapiro, 2019). In the presence of Cre recombinase, exons 2–4 of the *Kcnq2^f/f^* mice are excised, leading to a premature stop codon and a non-functional protein. C57BL/6J (RRID:IMSR_JAX:000664), B6;129S6-*Polr2a^Tn(pb-CAG-GCaMP5g,-tdTomato)Tvrd^*/J (RRID:IMSR_JAX:024477| PC-G5-tdT), and B6.129S2-*Emx1^tm1(cre)Krj^*/J (RRID:IMSR_JAX:005628| Emx1-Cre), were purchased from The Jackson Laboratory. Breeding was performed in-house at the University of Connecticut. For this study, we bred and developed *Emx1-cre::PC-G5-tdT::Kcnq2^f/+^* mice. We intercrossed these mice to obtain *Emx1-cre::PC-G5-tdT::Kcnq2^+/+^*, *Emx1-cre::PC-G5-tdT::Kcnq2^f/+^*, and *Emx1-cre::PC-G5-tdT::Kcnq2^f/f^* mice. For our experiments, we used either *Emx1-cre::PC-G5-tdT::Kcnq2^+/+^*or *Emx1-cre::PC-G5-tdT::Kcnq2^f/f^* mice. td-Tomato was used to verify that Cre expression was restricted to the forebrain. Annually we refresh the Cre-deleter line by purchasing new Emx1-cre breeding pairs and breeding them with a new set of mice carrying *Kcnq2^f/f^* and PC-G5-tdT. For *Emx1-cre::PC-G5-tdT::Kcnq3*^−/−^ we followed a similar strategy as with *Emx1-cre::PC-G5-tdT::Kcnq2^f/f^* line. *Kcnq3*^−/−^ mice were kept on a C57BL/6J background. Genotypes were confirmed with PCR. Experiments were performed on mice of either sex.

### Acute slice preparation

Mice between postnatal day (P)4 and P7 were brought from the vivarium into a designated area of the laboratory. Mice were then anesthetized using isoflurane. After verifying the mice were fully anesthetized, we rapidly decapitated them. Then, we placed the brains in ice-cold cutting solution containing the following: 26 mm NaHCO_3_, 210 mm sucrose, 10 mm glucose, 2.5 mm KCl, 1.25 mm NaH_2_PO_4_, 0.5 mm CaCl_2_, and 7 mm MgCl_2_. We mounted the brains on a vibratome (Leica, VS2000) and prepared 300 μm horizontal slices. Slices were then placed in a 35–37°C holding chamber for 30 min, and then moved to room temperature. The holding solution, which was the same as the recording solutions for the imaging and electrophysiological experiments, contained: 125 mm NaCl, 26 mm NaHCO_3_, 2.5 mm KCl, 1 mm NaH_2_PO_4_, 1.3 mm MgCl_2_, 1.5 mm CaCl_2_, and 12 mm D-glucose; 32–34°C, ∼310 mOsm. Slices were left to rest at room temperature for at least 1 h before experiments. All solutions were continuously bubbled with 5% CO_2_/95% O_2._ The slicing procedure was the same for whole-cell patch-clamp electrophysiology and calcium sensor imaging.

### Whole-cell patch-clamp electrophysiology

For electrophysiological recordings, we used an upright BX-51W Olympus microscope (Olympus). Neurons were visualized using the built-in Olympus microscope Nomarski optics, allowing for differential interference contrast. We recorded from pyramidal neurons of the CA3 region of the hippocampus. For current-clamp recordings we used borosilicate glass capillaries (WPI, TW150F-3). The pipette resistance was 3–4 MΩ when filled with a solution containing the following: 130 mm potassium methylsulfate, 10 mm KCl, 4 mm NaCl, 4 mm Mg·ATP, 0.4 mm Na_4_·GTP, 10 mm HEPES, and 5 mm Tris-phosphocreatine (osmolarity ∼300–310 mOsm). The pH was adjusted to 7.2–7.3 with KOH. Data were not corrected for the junction potential. All electrophysiological data were acquired using Multiclamp 700B amplifiers (Molecular Devices; RRID:SCR_018455) under the control of Clampex 10 software (Molecular Devices; RRID:SCR_011323). Data were acquired at 50 kHz and low-pass filtered at 10 kHz. For current-clamp recordings, the bridge balance circuit built into the Multiclamp 700B was engaged to correct for series resistance.

### Imaging acute slices

Optical imaging was performed on a Zeiss Axiozoom V.16 (Zeiss) using a PlanNeoFluar Z 1×, 0.25 NA objective with a 56 mm working distance. Images were recorded using an sCMOS camera (pco.edge 4.2) at a 500 × 500 pixel resolution. Image acquisition was performed using Micro-Manager 2.0 β (https://micro-manager.org/). Slices were submerged in a Warner chamber continuously perfused with extracellular solution (32–34°C).

### ΔF/F calculations for two-dimensional (2D) plots

We acquired and imported 300-s sCMOS sensor-recorded fluorescence time series (sampling rate: *F_s_* = 20 frames/s, 6000 frames/recording) in MATLAB (rel. 2019b; RRID:SCR_001622). Frames (500 × 500 pixels, interpixel distance: 6 μm) were inspected for the external edges of the area covered by each hemisphere, and only pixels within the area delineated by these lines were further considered. Pixels within this area were divided into *N* regions of interest (ROIs) using a simple linear iterative clustering (SLIC) algorithm (Achanta et al., 2012; MATLAB function: *superpixels*). The SLIC algorithm was applied to the first frame of each imported time series after foreground saturation to minimize the effects of spurious calcium activity at the beginning of the time series, and the value *N* was optimized by the algorithm using an unsupervised strategy. This resulted in ROIs that were spatially homogeneous, with pixels in each ROI showing similar fluorescence intensities. For any ROI n (n= 1, 2, …, *N*) and frame k (i.e., k= 1, 2, 3, …, 6000), the ROI intensity, $F_{n}\left(k\right)$, was estimated as the average value of the fluorescence intensity across all pixels in the ROI, and the series of variations in fluorescence intensity, $\Delta F_{n}/F$, was estimated according to the following formula:

(1) $$\frac{\Delta F_{n}}{F}\left(k\right)≜\frac{F_{n}\left(k\right)-\bar{F}\left(n,k\right)}{\bar{F}\left(n,k\right)},$$

where $\bar{F}\left(n,k\right)$ is the average value of $F_{n}\left(k\right)$ over a 30-s window centered on frame k. To ensure that $\bar{F}\left(n,k\right)$ captures the baseline background in the presence of sparse pulsatile activity, the computation of $\bar{F}\left(n,k\right)$ was as in Sood et al. (2019) and excluded values of $F_{n}\left(k\right)$ in the window above the 80th percentile. ROIs were numbered consecutively along the anteroposterior direction.

### Time-frequency analysis

A continuous wavelet transform was applied to every $\Delta F_{n}/F$ time series (analytic Morlet wavelet, 10 voices per octave), and the resultant scalograms $S_{n}\left(f,k\right)$ were used to detect transient and sustained oscillations in the ROIs. First, for any ROI n, the combinations (f,k) of frequency f and frame k at which the magnitude $\left|S_{n}\left(f,k\right)\right|$ was above a threshold $T_{1}$ identified *transient* oscillations (i.e., occurring at frame k) with period 1/f in the ROI. Second, for any ROI n, the cumulative magnitude ${\bar{S}}_{n}\left(f\right)=\underset{k}{\sum }\left|S_{n}\left(f,k\right)\right|$ was computed at all frequencies f, and the values $f^{*}$ at which ${\bar{S}}_{n}\left(f^{*}\right)$ was maximum and exceeded a threshold $T_{2}$ identified the presence of *sustained* activity (i.e., throughout the entire time series) at frequencies $f^{*}$ in the ROI. Threshold $T_{1}$ was set as the 95th percentile of the values $\left|S_{n}\left(f,k\right)\right|$ computed across all frequencies f, frames k, and ROIs n, and aimed to isolate the most prominent transient oscillations throughout the acute slice. Threshold $T_{2}$ was set as the 95th percentile of the values ${\bar{S}}_{n}\left(f\right)$ across all frequencies f and ROIs n. The combination of conditions (1) ${\bar{S}}_{n}\left(f^{*}\right)\geq {\bar{S}}_{n}\left(f\right)$ for all f and (2) ${\bar{S}}_{n}\left(f^{*}\right)>T_{2}$ guaranteed that only the most prominent sustained oscillations throughout the slice were retained.

Finally, the cumulative spectral content of every time series $\Delta F_{n}/F$ was computed in two separate frequency bands, i.e., 0.02–0.2 Hz (LF band) and 0.2–2.0 Hz (high-frequency band; HF). The spectral content, $P_{B}\left(n\right)$, of the series $\Delta F_{n}/F$ in the frequency band B (B=LF,HF) was computed as the integral of $\left|S_{n}\left(f,k\right)\right|$ over B and the number of frames, i.e.,

(2) $$P_{B}\left(n\right)=\frac{1}{F_{s}}\underset{k}{\sum }\overset{}{\underset{B}{\int }}\left|S_{n}\left(f,k\right)\right|df.$$

### ΔF/F calculations for pulse detection and measurements of amplitude and duration

ROIs exhibiting a pulsatile behavior were identified as follows. First, for any ROI n, the normalized series $\Delta F_{n}/F$ was evaluated for skewness (kurtosis value K≥4). If a series met the criteria for skewness, candidate peaks were selected by isolating local maxima with a minimum interpeak distance of 30 frames. Then, for each candidate peak, three measures were computed, i.e., peak prominence, peak width w, and root mean square (RMS) in the interval of length 2w centered on the peak. Candidate peaks were rejected if the prominence was lower than 3.5 or the RMS was lower than 2. Threshold values on prominence, RMS, and interpeak distance were determined offline based on the morphology and signal-to-noise ratio of the pulses in a sample series $\Delta F_{n}/F$. Finally, an ROI captured pulsatile activity if the corresponding time series $\Delta F_{n}/F$ retained three or more peaks after rejection. For ΔF/F measurements of amplitude, duration, and events/s, we used image files containing all 6000 frames imported to Fiji-ImageJ (https://imagej.net/Fiji; RRID:SCR_002285). To determine the ΔF/F for the anatomic regions representing the CA3 area of the hippocampus or the posterior, medial, or anterior cortex, we measured the fluorescence values by drawing a shell around these areas. Fluorescence data were then extracted using the image-stack-plot z built-in Fiji function. The data were then imported to AxoGraph ver. 1.7.5 (https://axograph.com/; RRID:SCR_014284) for automated event detection. The fluorescence changes (ΔF/F) were calculated by subtracting each data point from the mean of the baseline fluorescence values. ΔF/F amplitude (calcium events with amplitude ΔF/F > 0.01 were identified as events) and duration (50% of the event peak width) were analyzed using detection algorithms implemented in AxoGraph.

### Determination of wave propagation velocity

To assess the propagation of oscillations across the different ROIs, we tracked the migration of calcium waves through the fluorescence time series in each hemisphere. Specifically, for every time series $\Delta F_{n}/F$, we computed the envelope (i.e., absolute value of the analytic signal obtained via Hilbert transform) and retrieved the envelope’s highest peak, $p_{n}^{*}$. Then, we retained only those ROIs that (1) had a peak $p_{n}^{*}$ with prominence >3.5 and (2) presented a *sustained* oscillation, i.e., an oscillation with frequency $f^{*}$ as defined above, see Time-frequency analysis. Finally, the retained ROIs were sorted according to the arrival time, $t_{n}^{*}$, of $p_{n}^{*}$, and the propagation velocity was estimated for consecutive ROIs. Namely, denoted with $i_{1}$, $i_{2}$, $i_{3}$,…, the ROIs after sorting, we measured the propagation velocity between ROIs $i_{1}$ and $i_{2}$, ROIs $i_{2}$ and $i_{3}$, etc., according to the formula:

(3) $$v_{j}=\frac{\left\|C_{i_{j}}-C_{i_{j+1}}\right\|}{t_{i_{j+1}}^{*}-t_{i_{j}}^{*}},$$

where $v_{j}$ denotes the velocity in propagating from ROI $i_{j}$ (j= 1, 2, 3, …) to the next, $C_{i_{j}}$ is the centroid of the ROI $i_{j}$, and ‖·‖ denotes the Euclidian distance.

For each slice, the propagation velocity was reported as the average $v_{j}$ (mean ± SD) across the pairs of consecutive ROIs. If <7 values $v_{j}$ (i.e., eight ROIs) contributed to the propagation velocity, the slice was deemed not showing oscillatory activity, and no propagation velocity was computed. Also, if two or more ROIs had their peak $p_{n}^{*}$ arriving at the same time $t_{n}^{*}$, it was concluded that the ROIs were part of the same wavefront. Accordingly, the ROIs were preemptively combined into one region, and the average distance between the centroids of these ROIs and the centroid of the next ROI in the sequence was used in formula (3). The use of the Hilbert-transform and the signal envelope aimed to reduce the impact of noise on the time series and better capture the peak of the normalized fluorescence intensity, which was used as a proxy for a traveling wavefront. Also, the ROI aggregation and sorting procedures described above aimed to capture the spatial migration of a traveling wave.

### Local field potential (LFP) recordings

LFP recordings were taken in parallel with the imaging sessions. LFPs were recorded using an RHD2000 multi-channel amplifier (Intan Technologies) acquired with RHD2000 interface software (version 1.5, copyright Intan Technologies). LFP recordings were acquired at 20 kHz, low-pass filtered (eighth order Chebyshev Type I IIR filter with cutoff frequency at 80 Hz and zero-phase distortion), and downsampled to 200 Hz.

### Statistics

Statistical analyses were performed using GraphPad Prism 8 software (GraphPad; RRID:SCR_002798). Summary data are expressed as bar graphs, box plots, or violin plots. Box plots show the interquartile range extending from the 25th and 75th percentiles, whereas the box plot whiskers illustrate the minimum and maximum values. We used box plots to summarize the distribution of the results and to allow the reader to quickly compare data between conditions. Violin plots allow better visualization of the distribution of the data points than box plots; thus, we used violin plots to demonstrate the full effect of the loss of *Kcnq2* or *Kcnq3* on the amplitude, duration, and events/s of the calcium responses. For analysis, we used unpaired or paired Student’s *t* tests, Mann–Whitney *U* non-parametric unpaired tests, or Wilcoxon paired tests. Non-parametric tests were used for datasets in which the distribution of the data failed normality tests.

**Table 1:** Summary data and statistical analysis for **Figures 1-13**

Figures			Values			Statistical tests		
Figure 1
			Control	*Pyr:Kcnq2*	
1*B*	CA3	Duration	1.6 ± 0.46 s	2.2 ± 0.35 s	Mann–Whitney *U* test	*p* = 0.26	Control: *n* = 10; *Pyr:Kcnq2*: *n* = 18 hemispheres
		Events/s	0.0054 ± 0.0015	0.013 ± 0.0025	Mann–Whitney *U* test	*p* = 0.0143	Control: *n* = 22; *Pyr:Kcnq2*: *n* = 21 hemispheres
	Posterior cortex	Duration	1.15 ± 0.24 s	5.3 ± 0.61 s	Mann–Whitney *U* test	*p* < 0.0001	Control: *n* = 15; *Pyr:Kcnq2*: *n* = 20 hemispheres
		Events/s	0.008 ± 0.0018 Hz	0.015 ± 0.0018 Hz	Mann–Whitney *U* test	*p* = 0.0039	Control: *n* = 22; *Pyr:Kcnq2*: *n* = 20 hemispheres Animals: Control: *n* = 6; *Pyr:Kcnq2*: *n* = 6.
1*C*	CA3	APs vs current injections			Two-way ANOVA	(*p* = 0.0025; *F*_(9,33)_ = 3.745)	Control: *n* = 5; *pPyr:Kcnq2*: *n* = 6 cells
Figure 2
			Control	*Pyr:Kcnq2*	Statistical tests and values		
2*C*	CA3	Amplitude	0.064 ± 0.007 △F/F	0.14 ± 0.014 △F/F	Mann–Whitney *U* test	*p* < 0.0001	Control: *n* = 46; *Pyr:Kcnq2*: *n* = 56 hemispheres
		Duration	1.4 ± 0.18 s	8.5 ± 0.68 s	Mann–Whitney *U* test	*p* < 0.0001	
		Events/s	0.26 ± 0.023 Hz	0.043 ± 0.0047 Hz	Mann–Whitney *U* test	*p* < 0.0001
	Posterior cortex (PC)	Amplitude	0.08 ± 0.006 △F/F	0.29 ± 0.023 △F/F	Mann–Whitney *U* test	*p* < 0.0001	Control: *n* = 44; *Pyr:Kcnq2*: *n* = 56 hemispheres
		Duration	2.2 ± 0.19 s	6.5 ± 0.37 s	Mann–Whitney *U* test	*p* < 0.0001	
		Events/s	0.14 ± 0.0072 Hz	0.048 ± 0.0032 Hz	Mann–Whitney *U* test	*p* < 0.0001
	Medial cortex (MC)	Amplitude	0.021 ± 0.01 △F/F	0.33 ± 0.029 △F/F	Mann–Whitney *U* test	*p* < 0.0001	Control, *n* = 43; *Pyr:Kcnq2*: *n* = 55 hemispheres
		Duration	1.4 ± 0.58 s	9.5 ± 0.56 s	Mann–Whitney *U* test	*p* < 0.0001	
		Events/s	0.0065 ± 0.0024 Hz	0.018 ± 0.0023 Hz	Mann–Whitney *U* test	
	Anterior cortex (AC)	Amplitude	0.02 ± 0.003 △F/F	0.36 ± 0.03 △F/F	Mann–Whitney *U* test	*p* < 0.0001	Control: *n* = 44; *Pyr:Kcnq2*: *n* = 54 hemispheres
		Duration	1.5 ± 0.3 s	11.2 ± 0.8 s	Mann–Whitney *U* test	*p* < 0.0001	
		Events/s	0.012 ± 0.0021 Hz	0.021 ± 0.0025 Hz	Mann–Whitney *U* test	*p* = 0.0005
Data were obtained from 8 control and 9 *Pyr:Kcnq2 mice.*							
Figure 3
			Control	*Pyr:Kcnq2*	Statistical tests and values		
3*C*	Power 0.02–0.2 Hz		0.022 ± 0.0015 A.U.	0.11 ± 0.0046 A.U.	Mann–Whitney *U* test	*p* < 0.0001	Control, *n* = 43; *Pyr:Kcnq2*:, *n* = 47 hemispheres
	Power 0.2–2 Hz		0.026 ± 0.011 A.U.	0.38 ± 0.012A.U.	Mann–Whitney *U* test	*p* < 0.0001	
3*D*	ROI 0.02–0.2 Hz		10.1 ± 0.7 ROIs	19.9 ± 0.7 ROIs	Mann–Whitney *U* test	*p* < 0.0001	Control, *n* = 43; *Pyr:Kcnq2*:, *n* = 47 hemispheres
	ROI 0.2–2 Hz		6.6 ± 0.5 ROIs	0.5 ± 0.2 ROIs	Mann–Whitney *U* test	*p* < 0.0001	
Data were obtained from 8 control and 9 *Pyr:Kcnq2 mice.*							
Figure 4
				Control	*Pyr:Kcnq2*	Statistical tests and values		
4*C*	CA3	Amplitude	–PTX 0.057 △F/F ± 0.055 SD	–PTX 0.08 △F/F ± 0.09 SD	Mann–Whitney *U* test	Control *p* < 0.0001	Control: –PTX: *n* = 1547; +PTX: *n* = 1322 events *Pyr:Kcnq2*: –PTX: *n* = 224 events ; +PTX: *n* = 523 events
			+PTX 0.27 △F/F ± 0.12 SD	+PTX 0.19 △F/F ± 0.09 SD	Mann–Whitney *U* test	*Pyr:Kcnq2* *p* < 0.0001	
		Duration	–PTX 0.8 s ± 1.5 SD	–PTX 4.3 s ± 5.4 SD	Mann–Whitney *U* test	Control *p* < 0.0001
			+PTX 0.7 ± 1.7 SD	+PTX 1.8 ± 3.8 SD	Mann–Whitney *U* test	*Pyr:Kcnq2* *p* < 0.0001
	Posterior cortex	Amplitude	–PTX 0.08 △F/F ± 0.058 SD	–PTX 0.21 △F/F ± 0.26 SD	Mann–Whitney *U* test	Control *p* < 0.0001	Control: –PTX: *n* = 931; +PTX: *n* = 777 events *Pyr:Kcnq2*: –PTX: *n* = 223; +PTX: *n* = 444 events
			+PTX 0.43 △F/F ± 0.20 SD	+PTX 0.38 △F/F ± 0.24 SD	Mann–Whitney *U* test	*Pyr:Kcnq2* *p* < 0.0001	
		Duration	–PTX 1.9 s ± 2.4 SD	–PTX 5.1 s ± 4.5 SD	Mann–Whitney *U* test	Control *p* < 0.0001
			+PTX 0.58 s ± 1.0 SD	+PTX 1.5 ± 1.9 SD	Mann–Whitney *U* test	*Pyr:Kcnq2* *p* < 0.0001
	Medial cortex	Amplitude	–PTX 0.053 △F/F ± 0.066 SD	–PTX 0.21 △F/F ± 0.25 SD	Mann–Whitney *U* test	Control *p* < 0.0001	Control: –PTX: *n* = 94; +PTX: *n* = 270 events *Pyr:Kcnq2*: –PTX: *n* = 62; +PTX: *n* = 267 events
			+PTX 0.13 △F/F ± 0.22 SD	+PTX 0.27 △F/F ± 0.24 SD	Mann–Whitney *U* test	*Pyr:Kcnq2* *p* = 0.0003	
		Duration	–PTX 0.9 s ± 0.74 SD	–PTX 7.8 s ± 6.7 SD	Mann–Whitney *U* test	Control *p* < 0.0034
			+PTX 1.3 s ± 2.3 SD	+PTX 2.1 ± 3.3 SD	Mann–Whitney *U* test	*Pyr:Kcnq2* *p* < 0.0001
	Anterior cortex	Amplitude	–PTX 0.048 △F/F ± 0.038 SD	–PTX 0.16 △F/F ± 0.26 SD	Mann–Whitney *U* test	Control: *p* < 0.0001	Control: –PTX: *n* = 125; +PTX: *n* = 310 events *Pyr:Kcnq2*: –PTX: *n* = 150; +PTX: *n* = 66 events
			+PTX 0.14 △F/F ± 0.1 SD	+PTX 0.37 △F/F ± 0.31 SD	Mann–Whitney *U* test	*Pyr:Kcnq2* *p* < 0.0001	
		Duration	–PTX 1.0 s ± 1.3 SD	–PTX 4.8 s ± 6.1 SD	Mann–Whitney *U* test	Control *p* = 0.11
			+PTX 0.8 s ± 0.9 SD	+PTX 5.3 ± 7.1 SD	Mann–Whitney *U* test	*Pyr:Kcnq2* *p* = 0.87
Data were obtained from 3 control and 3 *Pyr:Kcnq2 mice.*							
Figure 5
			Control	*Pyr:Kcnq2*	Statistical tests and values		
5*C*	Power 0.02–0.2 Hz		–PTX 0.02 ± 0.003 A.U.	–PTX 0.11 ± 0.01 A.U.	Control Wilcoxon paired test	Control *p* < 0.0001	Control: *n* = 20; *Pyr:Kcnq2*: *n* = 14 hemispheres
			+PTX 0.06 ± 0.007 A.U.	+PTX 0.18 ± 0.01 A.U.	*Pyr:Kcnq2* paired Student’s *t* test	*p* < 0.0001 *t* = 6.233, df = 13	
	Power 0.2–2 Hz		–PTX 0.28 ± 0.02 A.U.	–PTX 0.37 ± 0.02 A.U.	Control Wilcoxon paired	Control *p* < 0.0001	Control: *n* = 20; *Pyr:Kcnq2*: *n* = 14 hemispheres
			+PTX 0.58 ± 0.03 A.U.	+PTX 0.73 ± 0.0 A.U.6	*Pyr:Kcnq2* Wilcoxon paired test	*Pyr:Kcnq2* *p* < 0.0001	
5*D*	ROI 0.02–0.2 Hz		–PTX 8.8 ± 0.7 ROIs	–PTX 23.0 ± 1.4 ROIs	Control paired Student’s *t* test	Control *p* = 0.84 *t* = 0.2042, df = 19	Control: *n* = 20; *Pyr:Kcnq2*: *n* = 14 hemispheres
			+PTX 9.1 ± 1.1 ROIs	+PTX 25.2 ± 1.4 ROIs	*Pyr:Kcnq2* paired Student’s *t* test	*Pyr:Kcnq2* *p* = 0.19 *t* = 1.377, df = 13
	ROI 0.2–2 Hz		–PTX 7.6 ± 0.6 ROIs	–PTX 0 ROI	Control Wilcoxon paired test	Control *p* = 0.72	Control: *n* = 20; *Pyr:Kcnq2*: *n* = 14 hemispheres
			+PTX 8.7 ± 1.6 ROIs	+PTX 0 ROI	*Pyr:Kcnq2* N/A	N/A
Data were obtained from 4 control and 3 *Pyr:Kcnq2 mice.*							
Figure 6
			Control	*Pyr:Kcnq2*	Statistical tests and values		
6*C*	CA3	Amplitude	–D-APV 0.070 △F/F ± 0.069 SD	–D-APV 0.13 △F/F ± 0.14 SD	Mann–Whitney *U* test	Control: *p* < 0.0001	Control: –D-APV *n* = 1265; +D-APV: *n* = 571 events *Pyr:Kcnq2*: –D-APV *n* = 193; +D-APV: *n* = 156 events
			+D-APV 0.036 △F/F ± 0.014 SD	+D-APV 0.09 △F/F ± 0.1 SD	Mann–Whitney *U* test	*Pyr:Kcnq2*: *p* < 0.0001	
		Duration	–D-APV 0.88 s ± 2.0 SD	–D-APV 5.4 s ± 5.8 SD	Mann–Whitney *U* test	Control: *p* < 0.0001
			+D-APV 0.78 ± 1.2 SD	+D-APV 5.4 ± 7.1 SD	Mann–Whitney *U* test	*Pyr:Kcnq2*: *p* = 0.0055
	Posterior cortex	Amplitude	–D-APV 0.07 △F/F ± 0.049 SD	–D-APV 0.26 △F/F ± 0.36 SD	Mann–Whitney *U* test	Control: *p* < 0.0001	Control: –D-APV *n* = 543; +D-APV *n* = 201 events *Pyr:Kcnq2*: –D-APV *n* = 243; +D-APV *n* = 170 events
			+D-APV 0.02 △F/F ± 0.01 SD	+D-APV 0.13 △F/F ± 0.14 SD	Mann–Whitney *U* test	*Pyr:Kcnq2*: *p* < 0.0001	
		Duration	–D-APV 1.99 s ± 2.1 SD	–D-APV 5.1 s ± 4.2 SD	Mann–Whitney *U* test	Control: *p* < 0.0001
				+D-APV 2.5s ± 2.7 SD	+D-APV 10.8 ± 6.6 SD	Mann–Whitney *U* test	*Pyr:Kcnq2*: *p* < 0.0001
	Medial cortex	Amplitude	–D-APV 0.11 △F/F ± 0.16 SD	–D-APV 0.18 △F/F ± 0.2 SD	Control: N/A	Control: N/A	Control –D-APV *n* = 10; +D-APV *n* = 0 events *Pyr:Kcnq2* –D-APV *n* = 158; +D-APV, *n* = 50events;
			+D-APV N/A	+D-APV 0.14 △F/F ± 0.16 SD	*Pyr:Kcnq2*: Mann–Whitney *U* test	*Pyr:Kcnq2*: *p* = 0.22	
		Duration	–D-APV 7.8s ± 8.4 SD	–D-APV 6.3 ± 6.2 SD	Control: N/A	Control: N/A
			+D-APV N/A	+D-APV 12.9 ± 8.4 SD	*Pyr:Kcnq2*: Mann–Whitney *U* test	*Pyr:Kcnq2*: *p* < 0.0001
	Anterior cortex	Amplitude	–D-APV 0.017 △F/F ± 0.006 SD	–D-APV 0.31 △F/F ± 0.4 SD	Control: N/A	Control: N/A	Control –D-APV *n* = 54; +D-APV *n* = 0 events *Pyr:Kcnq2*: –D-APV *n* = 71; +D-APV *n* = 29 events
			+D-APV N/A	+D-APV 0.15 △F/F ± 0.19 SD	*Pyr:Kcnq2*: Mann–Whitney *U* test	*Pyr:Kcnq2*: *p* = 0.0055	
		Duration	–D-APV 2.5 s ± 3.2 SD	–D-APV 8.9 s ± 7.1 SD	Control: N/A	Control: N/A
			+D-APV N/A	+D-APV 13.3 ± 11 SD	*Pyr:Kcnq2*: Mann–Whitney *U* test	*Pyr:Kcnq2*: *p* = 0.04
Data were obtained from 3 control and 3 *Pyr:Kcnq2 mice.*							
Figure 7
			Control	*Pyr:Kcnq2*	Statistical tests and values		
7*C*	Power 0.02–0.2 Hz		–D-APV 0.019 ± 0.0015 A.U.	–D-APV 0.11 ± 0.0085 A.U.	Control: Wilcoxon paired	Control: *p* < 0.0001	Control: *n* = 20; *Pyr:Kcnq2*: *n* = 14 hemispheres
			+D-APV 0.014 ± 0.0015 A.U.	+D-APV 0.069 ± 0.0057 A.U.	*Pyr:Kcnq2*: paired Student’s *t* test	*Pyr:Kcnq2*: *p* < 0.0001, *t* = 12.64, df = 13	
	Power 0.2–2 Hz		–D-APV 0.24 ± 0.012 A.U.	–D-APV 0.39 ± 0.022 A.U.	Control: Wilcoxon paired	Control: *p* = 0.0007	Control: *n* = 20; *Pyr:Kcnq2*: *n* = 14 hemispheres
			+D-APV 0.18 ± 0.017 A.U.	+D-APV 0.26 ± 0.0080 A.U.	*Pyr:Kcnq2*: Wilcoxon paired	*Pyr:Kcnq2*: *p* = 0.0001	
7*D*	ROI 0.02–0.2 Hz		–D-APV 8.9 ± 0.65 ROIs	–D-APV 25.21 ± 1.3 ROIs	Control: paired Student’s *t* test	Control: *p* = 0.20 *t* = 1.32, df = 19	Control: *n* = 20; *Pyr:Kcnq2*: *n* = 14 hemispheres
			+D-APV 7.5 ± 0.92 ROIs	+D-APV 20.71 ± 1.0 ROIs	*Pyr:Kcnq2*: paired Student’s *t* test	*Pyr:Kcnq2*: *p* = 0.0002 *t* = 5.22, df = 13	
	Power 0.2–2 Hz		–D-APV 7.05 ± 0.54 ROIs	–D-APV 0 ROIs	Control: paired Student’s *t* test	Control: *p* = 0.003 *t* = 3.472, df = 19	Control: *n* = 20; *Pyr:Kcnq2*: *n* = 14 hemispheres
			+D-APV 4.5 ± 0.54 ROIs	+D-APV 0.43 ± 0.2 ROIs	*Pyr:Kcnq2*: N/A	N/A	
Data were obtained from 3 control and 3 *Pyr:Kcnq2 mice.*							
Figure 8
P/N/A refers to Picrotoxin/APV/NBQX			Control	*Pyr:Kcnq2*	Statistical tests and values		
8*B*	CA3	Amplitude	–P/N/A: 0.046 Δ F/F ± 0.14 SD	–P/N/A:0.105 ΔF/F ± 0.14 SD	Mann–Whitney *U* test	Control: N/A	Control –P/N/A: *n* = 444; +P/N/A: *n* = 0 events *Pyr:Kcnq2*: –P/N/A: *n* = 246; +P/N/A: *n* = 38 events
			N/A	+P/N/A: ΔF/F 0.14 ± 0.13 SD		*Pyr:Kcnq2* *p* = 0.0001	
		Duration	–P/N/A: 0.59 s ± 1.3 SD	–P/N/A: 4.5 s ± 6.7 SD	Mann–Whitney *U* test	Control: N/A
			N/A	+P/N/A: 13.5 s ± 7.5 SD		*Pyr:Kcnq2*: *p* < 0.0001
	Posterior cortex	Amplitude	0.059 Δ F/F ± 0.03 SD	–P/N/A:0.48 Δ F/F ± 0.63 SD	Mann–Whitney *U* test	Control: N/A	Control –P/N/A: *n* = 228; +P/N/A: *n* = 0 events *Pyr:Kcnq2*: –P/N/A: *n* = 194; +P/N/A: *n* = 36 events
			N/A	+P/N/A: Δ 0.71 F/F ± 0.47 SD		*Pyr:Kcnq2*: *p* = 0.009	
		Duration	–P/N/A: 1.66 s ± 1.7 SD	–P/N/A: 4.6 s ± 3.9 SD	Mann–Whitney *U* test	Control: N/A
				N/A	+P/N/A: 10.2 s ± 3.6 SD		*Pyr:Kcnq2*: *p* < 0.0001
	Medial cortex	Amplitude	0.028 Δ F/F ± 0.018 SD	–P/N/A:0.38 Δ F/F ± 0.31 SD	Mann–Whitney *U* test	Control: N/A	Control –P/N/A: *n* = 22; +P/N/A: *n* = 0 events *Pyr:Kcnq2*: –P/N/A: *n* = 64; +P/N/A: *n* = 56 events
			N/A	+P/N/A: Δ 0.19 F/F ± 0.17 SD		*Pyr:Kcnq2*: *p* = 0.005	
		Duration	–P/N/A: 0.65 s ± 0.17 SD	–P/N/A: 7.6 s ± 4.7 SD	Mann–Whitney *U* test	Control: N/A
			N/A	+P/N/A: 8.9 s ± 7.5 SD		*Pyr:Kcnq2*: *p* = 0.087
	Anterior cortex	Amplitude	0.029 Δ F/F ± 0.026 SD	–P/N/A:0.47 Δ F/F ± 0.48 SD	Mann–Whitney *U* test	Control: N/A	Control –P/N/A: *n* = 22; +P/N/A: *n* = 0 events *Pyr:Kcnq2*: –P/N/A: *n* = 82; +P/N/A: *n* = 43 events
			N/A	+P/N/A: Δ 0.35 F/F ± 0.38 SD		*Pyr:Kcnq2*: *p* = 0.087	
		Duration	–P/N/A: 0.77 s ± 0.63 SD	–P/N/A: 7.7 s ± 5.8 SD	Mann–Whitney *U* test	Control: N/A
			N/A	+P/N/A: 14.1 s ± 8.7 SD		*Pyr:Kcnq2*: *p* = 0.016
Data were obtained from 3 control and 3 *Pyr:Kcnq2 mice.*							
Figure 9
P/N/A refers to Picrotoxin/APV/NBQX			Control	*Pyr:Kcnq2*	Statistical tests and values		
9*C*,*D*	Power 0.02–0.2 Hz		–P/A/N 0.018 ± 0.0007 A.U.	–P/A/N 0.13 ± 0.0094 A.U.	Control: Wilcoxon paired	Control: *p* < 0.0001	Control: *n* = 18; *Pyr:Kcnq2*: *n* = 14 hemispheres
			+P/A/N 0.01 ± 0.0006 A.U.	+P/A/N 0.068 ± 0.0083 A.U.	*Pyr:Kcnq2*: Wilcoxon paired	*Pyr:Kcnq2*: *p* = 0.0001	
	Power 0.2–2 Hz		–P/A/N 0.22 ± 0.007 A.U.	–P/A/N 0.45 ± 0.03 A.U.	Control: paired Student’s *t* test	Control: *p* < 0.0001 *t* = 9.106, df = 17	Control: *n* = 18; *Pyr:Kcnq2*: *n* = 14 hemispheres
			+P/A/N 0.13 ± 0.002 A.U.	+P/A/N 0.26 ± 0.01 A.U.	*Pyr:Kcnq2*: paired Student’s *t* test	*Pyr:Kcnq2*: *p* < 0.0001 *t* = 9.055, df = 13	
	ROI 0.02–0.2 Hz		–P/A/N 10.17 ± 0.93 ROIs	–P/A/N 19.86 ± 1.2 ROIs	Control: Wilcoxon paired	Control: *p* = 0.0001	Control: *n* = 18; *Pyr:Kcnq2*: *n* = 14 hemispheres
			+P/A/N 3.5 ± 0.85 ROIs	+P/A/N 15.14 ± 1.3 ROIs	*Pyr:Kcnq2*: paired Student’s *t* test	*Pyr:Kcnq2*: *p* = 0.0092 *t* = 3.057, df = 13	
	ROI 0.2–2 Hz		–P/A/N 8.7 ± 0.7 ROIs	–P/A/N 0 ROI	Control: Wilcoxon paired	Control: *p* < 0.0001	Control: *n* = 18; *Pyr:Kcnq2*: *n* = 14 hemispheres
			+P/A/N 0.72 ± 0.18 ROIs	+P/A/N 0 ROI	*Pyr:Kcnq2*: N/A		
Data were obtained from 3 control and 3 *Pyr:Kcnq2 mice.*							
Figure 10
			*Kcnq3^+/+^*	*Kcnq3*^−/−^	Statistical tests
10*C*	CA3	Amplitude	0.08 ± 0.005△F/F	0.08 ± 0.007△F/F	Welch’s *t* test	*p* = 0.95, *t* = 0.07052, df = 42	*Kcnq3^+/+^*, *n* = 23; *Kcnq3*^−/−^: *n* = 21 hemispheres
		Duration	2.4 ± 0.8 s	1.2 ± 0.2 s	Mann–Whitney *U* test	*p* = 0.93	
		Events/s	0.22 ± 0.03 Hz	0.19 ± 0.3 Hz	Mann–Whitney *U* test	*p* = 0.05	*Kcnq3^+/+^*, *n* = 24; *Kcnq3*^−/−^: *n* = 24 hemispheres
	Posterior cortex	Amplitude	0.073 ± 0.006△F/F	0.12 ± 0.01△F/F	Mann–Whitney *U* test	*p* < 0.0001	*Kcnq3^+/+^*, *n* = 23; *Kcnq3*^−/−^*^,^ n* = 24 hemispheres
		Duration	1.4 ± 0.3 s	4.5 ± 0.9 s	Mann–Whitney *U* test	*p* < 0.0001	
		Events/s	0.21 ± 0.025 Hz	0.12 ± 0.023 Hz	Mann–Whitney *U* test	*p* = 0.013	*Kcnq3^+/+^*, *n* = 24; *Kcnq3*^−/−^: *n* = 24 hemispheres
	Medial cortex	Amplitude	0.028 ± 0.008△F/F	0.09 ± 0.02△F/F	Mann–Whitney *U* test	*p* = 0.08	*Kcnq3^+/+^*, *n* = 13; *Kcnq3*^−/−^: *n* = 21 hemispheres
		Duration	3.2 ± 1.1 s	4.3 ± 1.2 s	Mann–Whitney *U* test	*p* = 0.004	
		Events/s	0.02 ± 0.12 Hz	0.017 ± 0.003 Hz	Mann–Whitney *U* test	*p* = 0.009	*Kcnq3^+/+^*, *n* = 24; *Kcnq3*^−/−^: *n* = 24 hemispheres
	Anterior cortex	Amplitude	0.03 ± 0.003△F/F	0.10 ± 0.13△F/F	Mann–Whitney *U* test	*p* < 0.0001	*Kcnq3^+/+^*, *n* = 20; *Kcnq3*^−/−^: *n* = 22 hemispheres
		Duration	3.1 ± 1.0 s	5.2 ± 1.3 s	Mann–Whitney *U* test	*p* = 0.72	
		Events/s	0.032 ± 0.007 Hz	0.05 ± 0.007 Hz	Mann–Whitney *U* test	*p* = 0.06	*Kcnq3^+/+^*, *n* = 24; *Kcnq3*^−/−^: *n* = 24 hemispheres
Data were obtained from 4 *Kcnq3^+/+^*and 4 *Kcnq3*^−/−^ *mice.*							
Figure 11
			*Kcnq3^+/+^*	*Kcnq3*^−/−^	Statistical tests
11*D*	Power 0.02–0.2 Hz		0.035 ± 0.005 A.U.	0.06 ± 0.005 A.U.	Mann–Whitney *U* test	*p* < 0.0001	*Kcnq3^+/+^*, *n* = 24; *Kcnq3*^−/−^: *n* = 24 hemispheres
	Power 0.2–2 Hz		0.57 ± 0.1 A.U.	0.53 ± 0.08 A.U.	Mann–Whitney *U* test	*p* = 0.67	
	ROI 0.02–0.2 Hz		12 ± 1 ROIs	25 ± 1 ROIs	Mann–Whitney *U* test	*p* < 0.0001	*Kcnq3^+/+^*, *n* = 24; *Kcnq3*^−/−^: *n* = 24 hemispheres
	ROI 0.2–2 Hz		6.2 ± 0.8 ROIs	0.6 ± 0.2 ROIs	Mann–Whitney *U* test	*p* < 0.0001	
Data were obtained from 4 *Kcnq3^+/+^*and 4 *Kcnq3*^−/−^ *mice.*							
Figure 12
P/N/A refers to Picrotoxin/APV/NBQX			*Kcnq3*^−/−^ –blockers	*Kcnq3*^−/−^ +blockers	Statistical tests		
12*B*	CA3	Amplitude	0.09 ± 0.009 △F/F	0.064 ± 0.008 △F/F	Student’s *t* test	*p* = 0.054, *t* = 2.007, df = 29	*Kcnq3*^−/−^: *n* = 16; +P/A/N: *n* = 15 hemispheres
		Duration (not illustrated)	1.1 ± 0.2 s	7.7 ± 1.6 s	Wilcoxon paired	*p* = 0.0006	*Kcnq3^-/^*: *n* = 16; P/A/N: *n* = 15 hemispheres
		Events/s	0.22 ± 0.03 Hz	0.019 ± 0.3 Hz	Wilcoxon paired test	*p* = 0.0002	*Kcnq3*^−/−^ *n* = 18; P/A/N: *n* = 18 hemispheres
	Posterior cortex	Amplitude	0.13 ± 0.01 △F/F	0.17 ± 0.02 △F/F	Wilcoxon paired	*p* = 0.15	*Kcnq3*^−/−^ *n* = 18; P/A/N: *n* = 15 hemispheres
		Duration (not illustrated)	3.1 ± 0.8 s	9.0 ± 0.9 s	Wilcoxon paired	*p* = 0.0006	*Kcnq3*^−/−^ *n* = 18; P/A/N: *n* = 15 hemispheres
		Events/s	0.16 ± 0.03 Hz	0.01 ± 0.003 Hz	Wilcoxon paired	*p* < 0.0001	*Kcnq3*^−/−^ *n* = 18; P/A/N: *n* = 18 hemispheres
	Medial cortex	Amplitude	0.12 ± 0.02△F/F	ND	N/D	N/D	*Kcnq3*^−/−^ *n* = 18; +P/A/N: *n* = 0 hemispheres
		Duration (not illustrated)	5.2 ± 1.6 s	ND	N/D	N/D	*Kcnq3*^−/−^ *n* = 18; P/A/N: *n* = 0 hemispheres
		Events/s	0.02 ± 0.004 Hz	ND	N/D	N/D	*Kcnq3*^−/−^ *n* = 18; P/A/N: *n* = 18 hemispheres
	Anterior cortex	Amplitude	0.1 ± 0.1△F/F	0.07 ± 0.02△F/F	Wilcoxon paired	*p* = 0.88	*Kcnq3*^−/−^ *n* = 18; P/A/N: *n* = 4 hemispheres
		Duration (not illustrated)	4.0 ± 0.7 s	9.6 ± 3.4 s	Wilcoxon paired	*p* = 0.25	*Kcnq3*^−/−^ *n* = 18; P/A/N: *n* = 4 hemispheres
		Events/s	0.06 ± 0.007 Hz	0.001 ± 0.0006 Hz	Wilcoxon paired	*p* < 0.0001	*Kcnq3*^−/−^ *n* = 18; P/A/N: *n* = 18 hemispheres
Data were obtained from 3 *Kcnq3^+/+^*and 3 *Kcnq3*^−/−^ *mice.*							
Figure 13
P/N/A refers to Picrotoxin/APV/NBQX			*Kcnq3^+/+^*	*Kcnq3*^−/−^	Statistical tests		
13*C*	Power 0.02–0.2 Hz		–P/A/N 0.025 ± 0.0019	–P/A/N 0.05 ± 0.003	*Kcnq3^+/+^*Wilcoxon paired	*p* < 0.0001	*Kcnq3^+/+^ n* = 17; *Kcnq3*^−/−^ *n* = 16 hemispheres
			+P/A/N 0.01 ± 0.0009	+P/A/N 0.02 ± 0.0018	*Kcnq3*^−/−^ paired Student’s *t* test	*p* < 0.0001 *t* = 9.531, df = 15
	Power 0.2–2 Hz		–P/A/N 0.33 ± 0.012	–P/A/N 0.33 ± 0.019	*Kcnq3^+/+^*paired Student’s *t* test	*p* < 0.0001 *t* = 11.60, df = 16
			+P/A/N 0.19 ± 0.006	+P/A/N 0.19 ± 0.011	*Kcnq3*^−/−^ paired Student’s *t* test	*p* < 0.0001 *t* = 8.787, df = 15
13*D*	ROI 0.02–0.2 Hz		–P/A/N 12.5 ± 0.96	–P/A/N 25 ± 1.3	*Kcnq3^+/+^*Wilcoxon paired	*p* = 0.0004 *t* = 4.477, df = 16	*Kcnq3^+/+^ n* = 17; *Kcnq3*^−/−^ *n* = 16 hemispheres
			+P/A/N 6.0 ± 1.1	+P/A/N 17.7 ± 1.6	*Kcnq3*^−/−^ paired Student’s *t* test	*p* = 0.0006 *t* = 4.321, df = 15	
	ROI 0.2–2 Hz		–P/A/N 7.1 ± 0.92	–P/A/N 0.75 ± 0.25	*Kcnq3^+/+^*Wilcoxon paired	*p* < 0.0001
			+P/A/N 0.76 ± 0.22	+P/A/N 0.81 ± 0.26	*Kcnq3*^−/−^Wilcoxon paired	*p* = 0.83
Data were obtained from 3 *Kcnq3^+/+^*and 3 *Kcnq3*^−/−^ mice							

## Results

KCNQ2 channels are expressed in neonatal excitatory neurons; however, it is currently unknown how KCNQ2 dysfunction alters forebrain network excitability early in development. To address this question, we investigated the role of KCNQ2 channels in controlling excitatory neural activity in developing neurons using *Kcnq2* conditional knock-out mice. To define the spatial and temporal pattern of excitatory neurons in acute slices and across the neocortex, we generated *Emx1^cre^::PC-G5-tdT::Kcnq2^f/f^* mice, denoted hereafter as *Pyr:Kcnq2* mice. In the presence of Cre recombinase, the *Pyr:Kcnq2* mice express the genetically encoded calcium sensor GCamp5g (Gee et al., 2014), which allowed us to visualize calcium events. Additionally, these mice express td-Tomato, enabling us to determine the extent of Cre recombinase activity and specificity. To maintain the circuitry between regions, we used horizontal slices from neonatal mice (P4–P7). We focused on this developmental age range because it is a critical period for the development and maturation of synaptic connectivity, corresponding to a prenatal period in humans.

### *Pyr:Kcnq2* neonatal slices exhibit increased excitability

**Figure 1.**
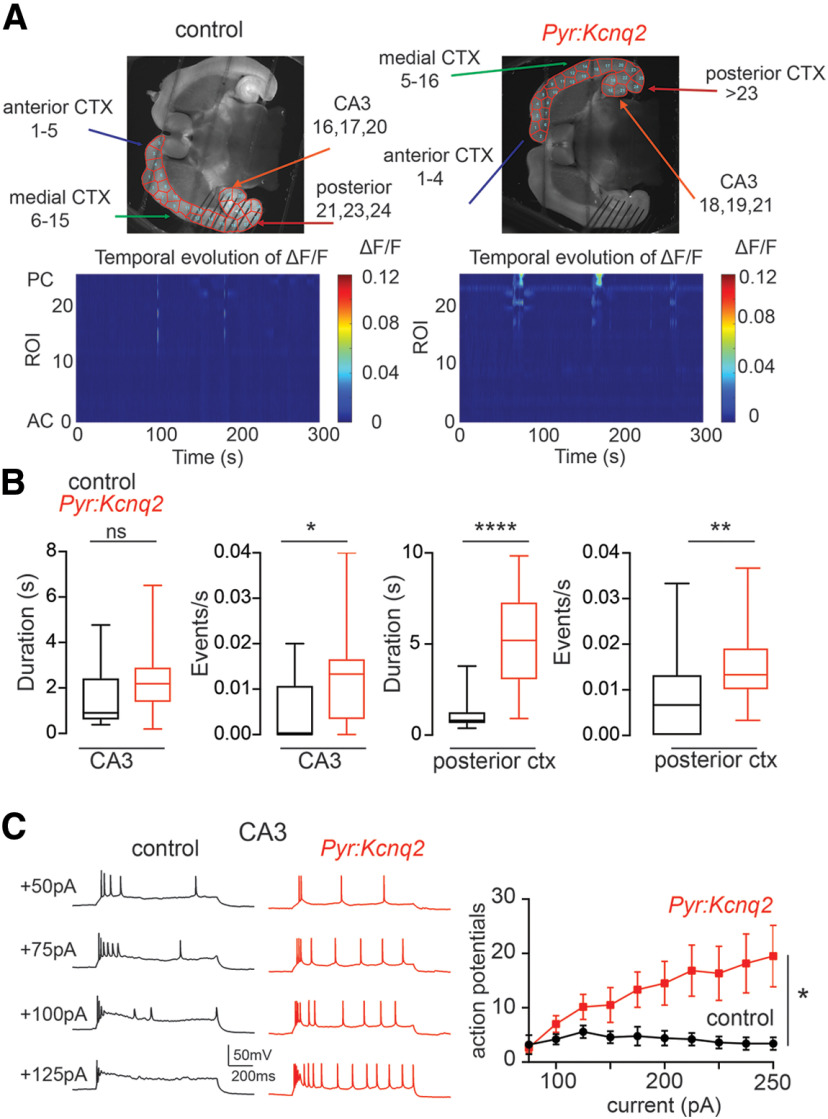
Ablation of *Kcnq2* from neonatal excitatory neurons leads to increased excitability in the hippocampus and posterior cortex. All recordings were in the presence of 2.5 mm Ko. ***A***, top panels, Examples of acute slices from control and *Pyr:Kcnq2* mice with one hemisphere segmented into ROIs. Bottom panels, Representative 2D plots showing calcium activity (ΔF/F) as a function of time for the different ROIs. The numbering corresponds to the segmented area shown on the top panels with lower values toward the anterior cortex (AC) and higher values toward the CA3 area of the hippocampus and posterior cortex (PC). ***B***, Summary graphs show the effect of *Kcnq2* ablation on the calcium event duration and frequency for the CA3 area of the hippocampus and the posterior cortex. Data in the box plots show the median and the interquartile range. (*p < 0.05, **p < 0.01, ****p < 0.0001). ***C***, Whole-cell recordings from CA3 pyramidal neurons show that ablation of *Kcnq2* increases neuronal excitability. Left panels, Family of depolarizing 1-s current steps in either control or *Pyr:Kcnq2* slices. Right, Summary graph shows the number of recorded action potentials in control and *Pyr:Kcnq2* CA3 pyramidal neurons. *p < 0.05 compared with control group. For ***C***, data are represented as mean ± SEM. Additional details on the statistical analysis for this figure are found in Table 1 under the Figure 1 section.

Figure 1*A* compares the widefield calcium activity in horizontal slices from control and *Pyr:Kcnq2* mice. In slices of both groups, the area covering each hemisphere was decomposed into ROIs using the SLIC clustering algorithm, which aggregates pixels in a ROI if they are spatially contiguous and of similar fluorescence intensity. In this way, ROIs were constructed as macro-areas that have similar fluorescence intensities across neighbor pixels, which assures anatomic homogeneity within each macro-area. Also, because of the intra-ROI homogeneity, the fluorescence signal was averaged across pixels in each ROI, thus reducing the effects of noise on the analysis of the fluorescence time series.

The results of the ROI partition indicate that *Pyr:Kcnq2* slices and control slices had a similar number of ROIs (31 ± 0.5 ROIs, *n* = 52 hemispheres vs 31 ± 0.5 ROIs, *n* = 64 hemispheres; *Pyr:Kcnq2* vs control *p* = 0.62, Student’s *t* test; *t* = 0.4971, df = 114) and ROIs of similar size (number of pixels per ROI: 971 ± 12 pixels, *n* = 52 hemispheres vs 963 ± 9 pixels, *n* = 64 hemispheres; *Pyr:Kcnq2* vs control *p* = 0.91, *t* = 0.4971, df = 114 Student’s *t* test). Thus, we observed no significant anatomic or functional differences between the two groups of slices.

Previous work has shown that spontaneous neuronal activity is infrequent and primarily confined to the hippocampal formation and entorhinal cortex in neonatal slices (Garaschuk et al., 2000; Namiki et al., 2013). Indeed, during a 5-min recording from control littermate slices, we observed calcium activity in the CA3 region of the hippocampus and the posterior cortex, which primarily represented the entorhinal cortex (Fig. 1*A*, bottom panels). As observed in previous studies, the activity was modest in intensity, with brief pulsatile periods and few events/s (Fig. 1*B*).

Next, we examined slices from mice lacking *Kcnq2* in excitatory cells. We note that the *Emx1-cre* mice express Cre recombinase as early as E10.5 (Gorski et al., 2002), thus, by P4, KCNQ2 levels in the majority of excitatory cells would be minimal. Consistent with this assertion, we found CA3 pyramidal neurons from *Pyr:Kcnq2* mice fired a greater number of action potentials following depolarizing current injections (Fig. 1*C*). Furthermore, in the absence of *Kcnq2*, calcium responses remained confined to the same pacemaker regions as in slices from control littermate mice, i.e., the CA3 region of the hippocampus and the posterior cortex (Fig. 1*A*). However, because of the loss of *Kcnq2*, the number of events during the recording period was significantly higher in both regions (0.012–0.015 events/s; approximately four to five events in 5 min; Fig. 1*B*). In addition to identifying changes in the number of detected events, we found that the duration but not amplitude of the calcium events in the posterior cortex was greater in the knock-out mice (control: 0.073 ± 0.014 ΔF/F, *n* = 14 hemispheres; *Pyr:Kcnq2*: 0.016 ± 0.03 ΔF/F, *n* = 20 hemispheres; *p* = 0.08 Mann–Whitney *U* test; Fig. 1*B*). By contrast, we observed no changes in the amplitude or duration of the calcium signal for the CA3 region of the hippocampus (control: 0.031 ± 0.006 ΔF/F, *n* = 9 hemispheres; *Pyr:Kcnq2*: 0.034 ± 0.005 ΔF/F, *n* = 18 hemispheres; *p* = 0.97 Mann–Whitney *U* test; Fig. 1*B*). The observed differences between the CA3 region of the hippocampus and the posterior cortex suggested that deletion of *Kcnq2* manifests in a distinct manner in different macro-circuits.

As with the slices from control animals, we did not detect any consistent large calcium transients during the 5-min recording window in the neocortex. Similarly, earlier work showed that overall cortical activity in acute slices is very low at this developmental time point (Garaschuk et al., 2000). We note that our fluorescence signal represented calcium activity across all neuronal subcompartments; thus, at this resolution, we could not determine whether the changes in calcium signals were because of increased excitability at dendrites, soma, or axons. These changes were most likely because of a combination of all three subcompartments, as KCNQ2 channels are not simply restricted to axons in the early developmental period.

**Figure 2.**
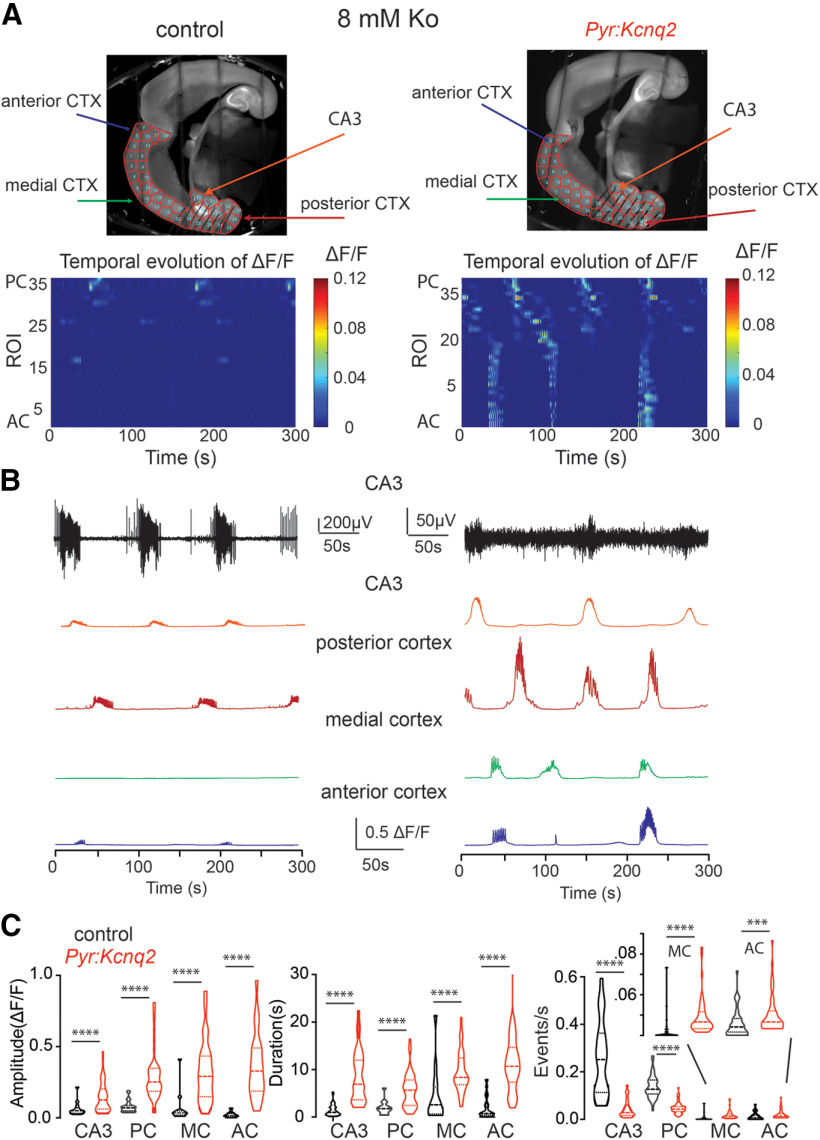
Deletion of *Kcnq2* from excitatory neurons leads to elevated calcium activity across the forebrain in 8 mm Ko. ***A***, top panels, Examples of acute slices from control and *Pyr:Kcnq2* mice with one hemisphere segmented into ROIs. Bottom panels, 2D plots show the calcium activity across the different ROIs. The numbering corresponds to the segmented area shown on the top panels with lower values toward the AC and higher values toward the PC. Note that in the absence of *Kcnq2*, substantial calcium activity is measured across all regions of the forebrain. ***B***, top two panels, Temporal evolution of the LFPs and ΔF/F recorded in parallel in the CA3 region of the hippocampus. Note that in contrast to the LFPs in slices from *Pyr:Kcnq2* mice, the calcium responses are large and long lasting. Middle and bottom panels, Temporal evolution of the ΔF/F across multiple ROIs. ***C***, Violin plots show the effect of *Kcnq2* deletion on the amplitude, duration, and frequency of the calcium events for different anatomic regions. MC refers to the medial cortex. Note that ablation of *Kcnq2* led to a large and uniform increase of the calcium response amplitude and duration. ****p < 0.0001. Additional details on the statistical analysis and number of replicates for this figure are found in Table 1 under the Figure 2 section.

To further probe the effects of *Kcnq2* deletion from the neocortex under more excitable conditions, we imaged slices that had been incubated with 8 mm extracellular potassium (Ko) for ∼20 min. The use of 8 mm Ko allowed us to compare the activity of different regions under constant stimulation conditions. A concentration of 8 mm Ko was necessary to increase the event number in our slices (Fig. 2). We applied 8 mm Ko to slices for no more than 20 min to prevent the slices from becoming epileptic (i.e., from exhibiting spontaneous ictal events following removal of the high-potassium solution). After incubating the slices with 8 mm Ko, we recorded 5-min time-lapse movies. To complement our calcium imaging, we also recorded LFPs from the CA3 region of the hippocampus, which allowed us to correlate calcium activity to the overall network activity. Similar to the recordings in low-potassium artificial cerebrospinal fluid, we found that the majority of calcium events in slices from control animals originated from the hippocampal formation and posterior cortex, closely following the measured LFPs (Fig. 2*B*; Movie 1).

In contrast to the control slices, slices from mice lacking KCNQ2 channels exhibited widespread calcium activity spanning all cortical regions and layers (Fig. 2; Movie 2). Closer examination of the time-lapse movies also showed that activity in the neocortex frequently initiated in the upper cortical layers and then migrated to the deeper layers and neighboring neocortical regions (see Movie 2). Calcium waves propagated in multiple directions and were generally multifocal, as the initial activity sprang at multiple regions. We also found that the calcium events were larger and longer lasting across all regions (Fig. 2*C*). Additionally, the average propagation velocity of the calcium responses across ROIs in slices from *Pyr:Kcnq2* mice was 0.64 ± 0.074 mm/s (*n* = 55 hemispheres), which was almost three times slower than the rate measured in control slices (2.07 ± 0.53 mm/s, *n* = 18 hemispheres, *p* = 0.0006 Mann–Whitney *U* test). We currently do not know the reason for this difference, but there could be many contributing factors such as the location of the calcium signal, or the presence of multiple waves with different latencies instead of one large, synchronous wave. We did not investigate this further.

Although the calcium activity was robust in CA3, we noticed that the LFPs were typically smaller or absent in slices from *Pyr:Kcnq2* mice. This result could be attributed to several reasons, such as electrode placement, increased GABAergic receptor activity (see below), the higher tendency of *Kcnq2*-null excitatory neurons to show spreading depression and persistent depolarization leading to sodium channel inactivation (Aiba and Noebels, 2021), or a combination of these mechanisms. We did not pursue this question further. Importantly, independent of the region being imaged, we observed an increase in both the amplitude and duration of calcium events (Fig. 2*C*). This trend was particularly striking in the medial and anterior cortex, where the event amplitude increased by ∼50-fold (i.e., medial cortex: from 0.02 ΔF/F to 0.33 ΔF/F).

In slices from *Pyr:Kcnq2* mice, large calcium events in CA3 and the posterior cortex were organized in LF patterns. Additionally, the average number of events in the calcium fluorescence signals in both regions was decreased compared with the number of events from control slices (CA3, decreased from 0.025 to 0.004 events/s; posterior cortex, decreased from 0.013 to 0.005 events/s; Fig. 2*C*). This shift in the number of events reflects the loss of small events and the emergence of longer-lasting less frequent large population events. Indeed, the duration of average calcium events increased from 1.4 to 8.5 and 2.2 to 6.5 s in the CA3 region of the hippocampus and the posterior cortex, respectively. In contrast, in the medial and anterior cortex, we primarily observed an increase rather than a decrease in the number of calcium events (medial cortex: 0.006 to 0.018 events/s; anterior cortex: 0.012 to 0.021 events/s; Fig. 2*C*), which primarily reflects the appearance of prolonged synchronous activity following the large depolarization of the posterior cortex and the CA3 region of the hippocampus. Thus, based on these data, we conclude that the loss of *Kcnq2* from excitatory neurons increases excitability, manifested as large population events, across the forebrain, independent of the region. This trend is consistent with the wide distribution of KCNQ2 channels in the forebrain (Cooper et al., 2001).

### *Kcnq2* deletion leads to transient slow-wave patterns

**Figure 3.**
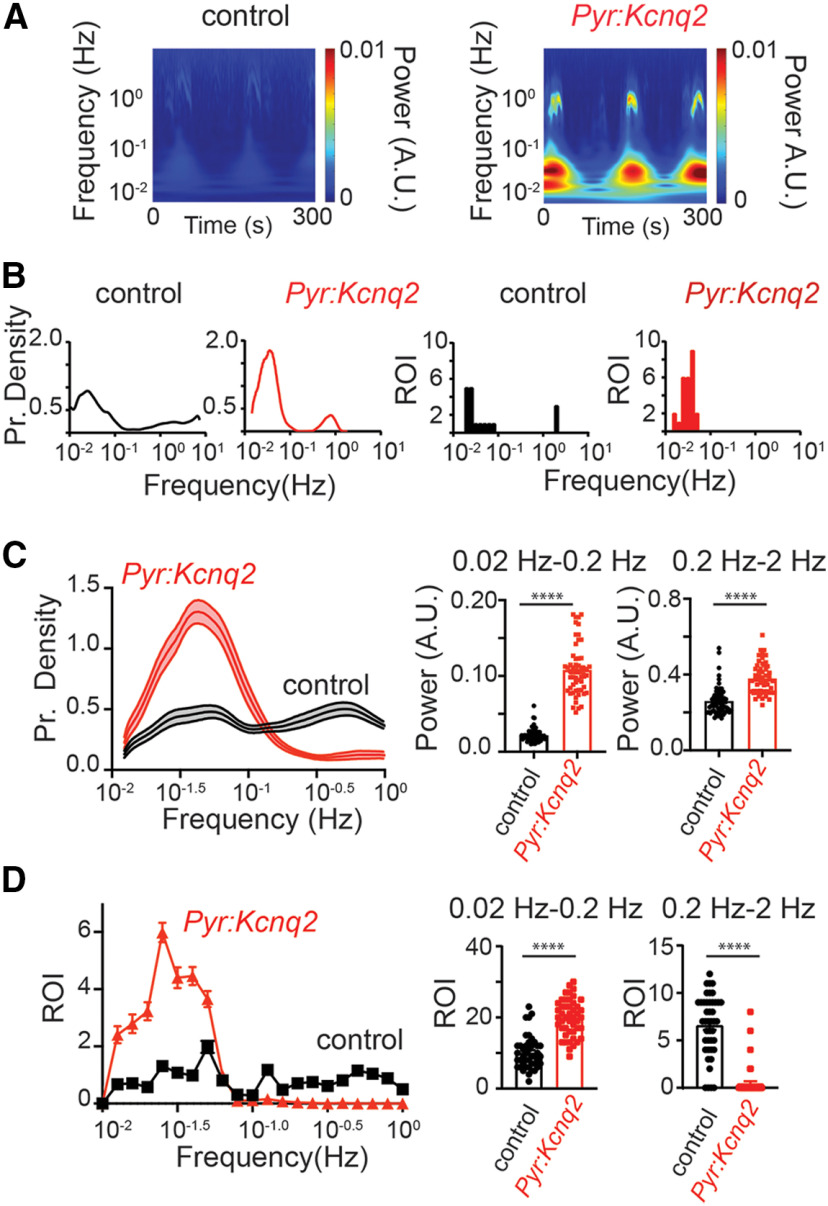
Deletion of *Kcnq2* leads to LF calcium oscillations. Deletion of *Kcnq2* increased the likelihood of LF oscillations across a large number of ROIs; oscillations were sustained throughout the duration of the recording period. All recordings were in the presence of 8 mm Ko. ***A***, Wavelets from control and *Pyr:Kcnq2* hemispheres. The wavelets were generated from the examples shown in Figure 2*A*. Note the large increase in the power for the oscillations occurring at the LF range in the hemisphere from *Pyr:Kcnq2* animals. Although in this example some increases in the power were also observed at the higher-frequency values, this was not seen across all slices, as shown in panel ***C***, right panels. ***B***, Comparison of the probability density of the frequency f of transient oscillations (left panels) and the number of ROIs undergoing sustained oscillations (right panels) for the examples depicted in panel ***A***. ***C***, left, Comparison of the probability density of the transient oscillations for control (*n* = 43) and *Pyr:Kcnq2* hemispheres (*n* = 47) across multiple slices. Data are represented as mean ± SEM. Middle and right panels, Summary graphs quantifying the power measured for the two frequency domains, 0.02–0.2 and 0.2–2 Hz. Note that loss of *Kcnq2* increased the likelihood of observing LF calcium oscillations ranging primarily from 0.03 to 0.05 Hz. Data are represented as mean ± SEM (****p < 0.0001). ***D***, left, Comparison of the ROIs undergoing sustained oscillations for control (*n* = 43) and *Pyr:Kcnq2* (*n* = 47) hemispheres. Results are reported for sustained oscillations whose frequency $f^{*}$ is in the LF and the HF range, respectively. See Materials and Methods, Time-frequency analysis, for a definition of the frequency $f^{*}$ of a sustained oscillation. Middle and right panels, Summary graphs of the ROIs at two frequency domains, 0.02–0.2 and 0.2–2 Hz (****p < 0.0001). Note that deletion of *Kcnq2* leads to a greater number of forebrain regions that show sustained LF calcium oscillations (from 0.012 to 0.05 Hz). This is in contrast to control slices that exhibit oscillation frequencies across a wider range of frequencies (from 0.012 to 1.2 Hz). Additional details on the statistical analysis and number of replicates for panels ***C***, ***D*** are found in Table 1 under the Figure 3 section.

To further quantify the effects of *Kcnq2* deletion on the calcium signals from excitatory cells, we conducted a time-frequency analysis of the fluorescence time series (Fig. 3) and measured the cumulative magnitude of these signals in the LF (0.02–0.2 Hz) and HF (0.2–2 Hz) bands, which captured the majority of the activity in our slices. Figure 3*A* shows the time-frequency analysis averaged across all ROIs and indicates that the loss of *Kcnq2* primarily increased activity in the LF band. Moreover, Figure 3*B*, left panels, shows the sample distribution of the frequency f of the transient oscillations across all ROIs for *Kcnq2* (red line) and control (black line), respectively, whereas Figure 3*B*, right panels, reports the number of ROIs that exhibited sustained oscillations (i.e., a histogram of frequencies $f^{*}$). All panels refer to the slices in Figure 2*A*. Figure 3*A*,*B* shows that deleting *Kcnq2* is associated with widespread oscillations at frequencies concentrated in the LF band. This result was confirmed at the population-level, i.e., we reported that, across all slices, the oscillations spanned a larger number of ROIs in *Pyr:Kcnq2* slices than control slices (Fig. 3*C*,*D*), were sustained throughout the duration of the recording period, and exhibited transient modulations of the fundamental frequency, although the fundamental frequency remained primarily confined to the LF band (Fig. 3*C*,*D*). In contrast, few ROIs showed significant periodic oscillations in the HF range, as summarized in Figure 3*D*.

Together, these findings are consistent with our earlier observation that loss of *Kcnq2* primarily leads to the appearance of large but less frequent population events (see Fig. 2). However, these data also show that deletion of *Kcnq2* from forebrain excitatory neurons leads to multiple hot spots at low frequencies in the presence of elevated extracellular potassium. These hot spots are distributed across the entire slice and can be spatially separated but retain similar frequencies, which strongly indicates that the hot spots could stem from increased network signaling.

### GABAA receptor activity amplifies without shaping *Kcnq2* slow-wave patterns

**Figure 4.**
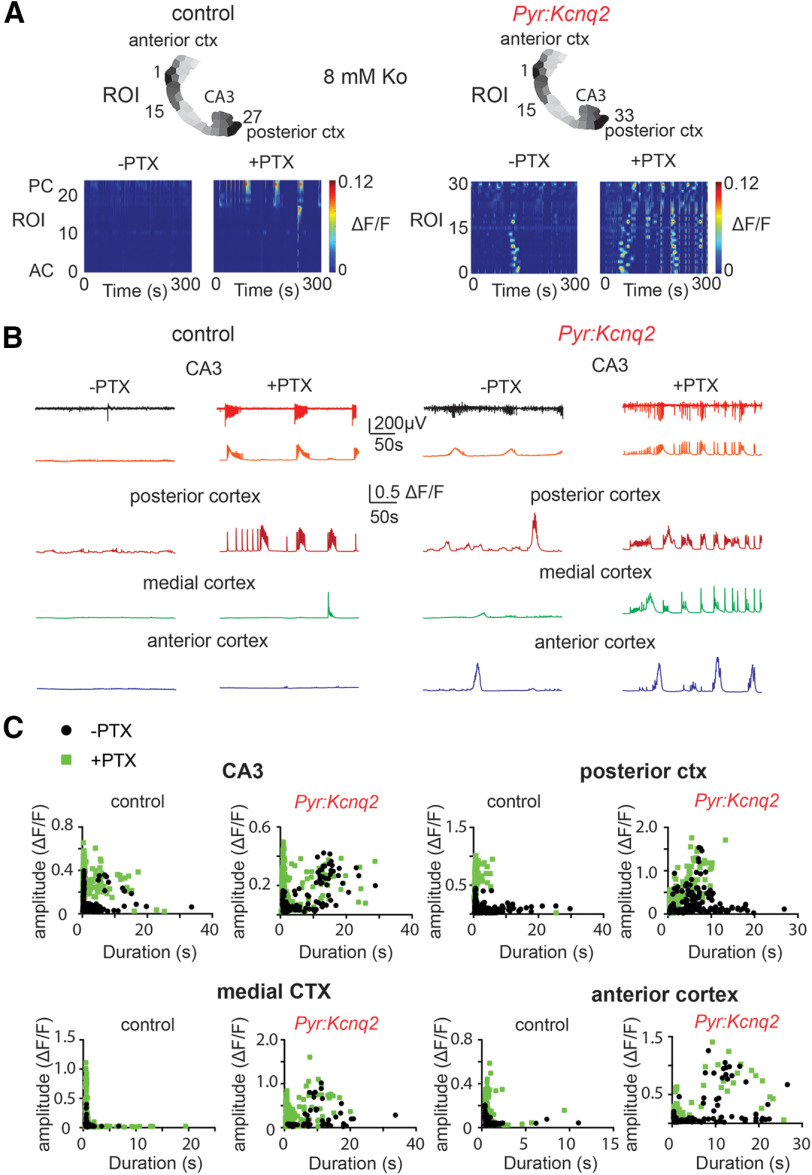
GABAA receptors limit activity in neonatal control and *Pyr:Kcnq2* brain slices. All recordings were in the presence of 8 mm Ko. ***A***, Panels show examples of acute slices from control and *Pyr:Kcnq2* mice before and after application of 50 μm PTX to block GABAA receptors. Top panels, Hemispheres segmented into ROIs. Bottom panels, 2D plots representing the changes in the calcium activity (ΔF/F) across the different ROIs on application of PTX. The numbering corresponds to the segmented area shown on top with lower values toward the AC and higher values toward the PC. Note that inhibiting GABAA receptors led to widespread calcium activity in the *Pyr:Kcnq2* hemisphere. In contrast, the activity in the control hemisphere was primarily confined to the posterior cortex and the hippocampal formation following application of PTX. ***B***, Calcium responses from the different anatomic regions before and after application of PTX. Note that in *Pyr:Kcnq2* slices application of PTX led to the appearance of a barrage of activity across all regions. ***C***, Scatter plots show the effect of PTX on the ΔF/F amplitude and duration. Note that application of PTX increased the number of larger and faster calcium signals. Additional details on the statistical analysis and number of replicates for this figure are found Table 1 under the Figure 4 section.

**Figure 5.**
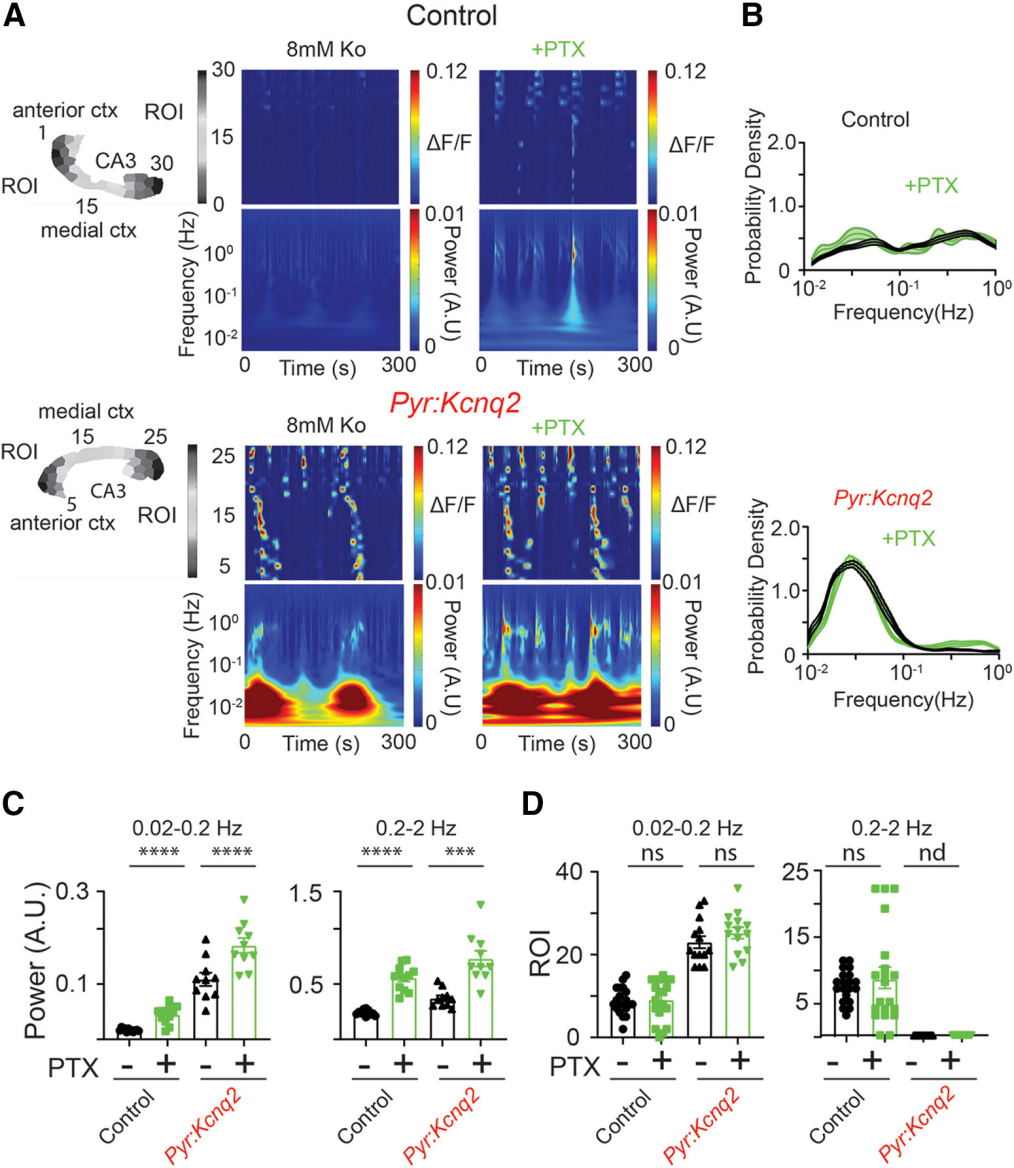
GABAA receptor activity limits the power but not the occurrence of LF calcium oscillations in *Pyr:Kcnq2* slices. All recordings were in the presence of 8 mm Ko. ***A***, Representative examples show calcium activity (ΔF/F) over time and the corresponding wavelets in the presence and absence of 50 μm PTX for control and *Pyr:Kcnq2* slices. Note the large increase in power at the LF range in *Pyr:Kcnq2* slices, further quantified across multiple slices in panel ***C***. ***B***, Comparison of the probability density of the frequency f for transient oscillations before and after PTX in control (*n* = 20) and *Pyr:Kcnq2* (*n* = 14) hemispheres. Data are represented as mean ± SEM. The absence of change in the probability density on blocking GABAA receptor activity suggests that GABAA receptors alter the peak oscillation frequency at the different ROIs. ***C***, Summary graphs show that application of PTX increased the power for the LF and HF domains in control and *Pyr:Kcnq2* slices. ***D***, Summary graphs show that on application of PTX the number of ROIs undergoing sustained oscillations in control and *Pyr:Kcnq2* slices did not change. Together, ***C***, ***D*** suggest that blocking GABAA receptors primarily increases the activity within each ROI. Data are presented as mean ± SEM (***p < 0.001, ****p < 0.0001). Additional details on the statistical analysis for panels ***C***, ***D*** are found in Table 1 under the Figure 5 section. nd, not determined.

To further understand the role of network signaling in the formation of LF hot spots, we conducted follow-up experiments to determine whether these slow oscillations are driven by glutamatergic or GABAergic activity. Figure 4 shows that inhibition of GABAA receptors using 50 μm picrotoxin (PTX), a ubiquitous GABAA receptor open channel antagonist that blocks both synaptic and extra-synaptic GABAA receptors, further exacerbated the increase in network activity in both control and *Pyr:Kcnq2* slices (Fig. 4*A*,*B*). Picrotoxin also sped up, almost doubling, the propagation velocity of the calcium activity in *Pyr:Kcnq2* slices (from 0.59 ± 0.11 to 1.08 ± 0.15 mm/s, *n* = 20 hemispheres, *p* = 0.0136 Wilcoxon pair test), further suggesting that GABAA receptors limit the spread of calcium responses in the forebrain. Our results are consistent with several studies showing that the application of PTX can increase network activity in the neonatal brain, even when GABA acts as an excitatory transmitter because of its depolarized chloride equilibrium potential in neonatal excitatory neurons (Kirmse et al., 2015; Che et al., 2018), but also see (Murata and Colonnese, 2020). Independent of the genotype, blocking GABAA receptors led to larger fluorescence signal amplitudes in all areas of the forebrain (Fig. 4*B*,*C*). However, the average calcium event duration decreased as blocking GABAA receptors led to the appearance of larger but briefer calcium signals in the *Pyr:Kcnq2* slices (Fig. 4*B*,*C*). We also observed this trend in the LFP recordings (Fig. 4*B*), suggesting that GABAA receptor activation dampens ongoing network activity in the absence of *Kcnq2* across the forebrain. Consistent with uniform upregulation of activity in slices from *Pyr:Kcnq2* mice, we found a generalized increment of the time-frequency signal in response to PTX independent of the genotype (Fig. 5*A*). This resulted in a significant increase of the power both in the LF and HF bands at the population-level (i.e., across slices), and the percent increase in power with respect to the pre-PTX case was similar in slices from *Pyr:Kcnq2* and control mice. vice versa, the probability density of the frequency f of transient oscillations in a slice (Fig. 5*B*,*C*) and the total number (i.e., across all slices) of ROIs exhibiting a sustained oscillation (Fig. 5*D*) were similar before and after applying PTX, which indicates that the temporal pattern of neural activity was not altered by PTX. Moreover, no ROIs in slices from *Pyr:Kcnq2* mice exhibited sustained oscillations in the HF band, and this trend remained unaffected by the application of PTX (Fig. 5*D*).

Combined, Figures 4, 5 indicate that PTX increases the energy of the fluorescence signals from each ROI without altering the temporal pattern of the activity, both in slices from *Pyr:Kcnq2* and control mice. This finding suggests that the application of PTX facilitates the synchronization among neurons within each ROI (i.e., local microcircuits) but has limited impact on the recruitment of distinct ROIs into a common pattern. It also suggests that the shift in behavior observed in slices from *Pyr:Kcnq2* mice is not affected by local synaptic GABAergic transmission but rather stems from a change in neural excitability throughout the forebrain.

### NMDA receptor inhibition attenuates *Kcnq2* slow-wave patterns

We then assessed whether the LF activity observed in slices from *Pyr:Kcnq2* mice depends on glutamatergic activity. Previous work has shown that in the presence of 8 mm Ko, increased network excitability is partly driven by the activation of NMDA glutamate receptors (Traynelis and Dingledine, 1988). This phenomenon occurs because NMDA receptors have slow deactivation kinetics, which allows for prolonged dendritic depolarization, leads to a downstream increase in somatic and axonal excitability, and causes a buildup of extracellular potassium.

**Figure 6.**
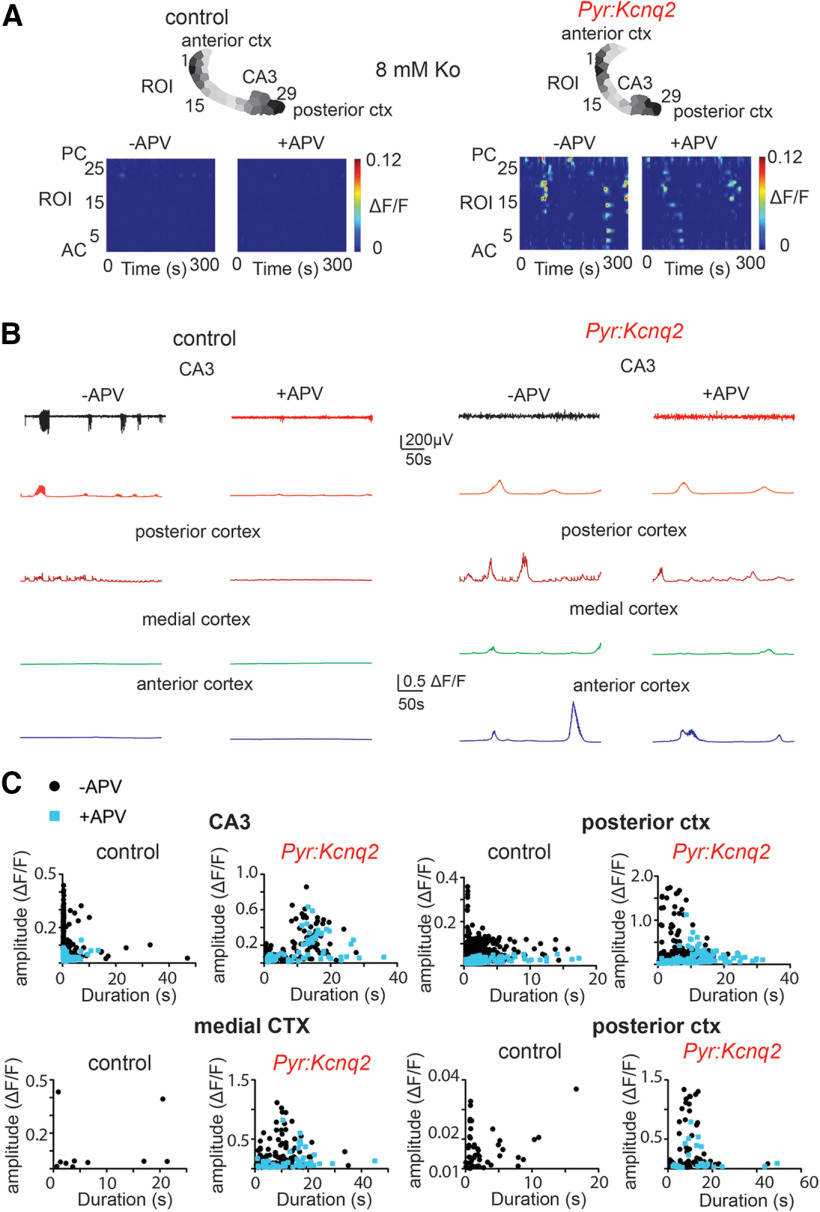
*Kcnq2* deficient slices acquired NMDA receptor independent calcium activities. All recordings were in the presence of 8 mm Ko. ***A***, Panels show examples of acute slices from control and *Pyr:Kcnq2* mice before and after application of 25 μm D-APV (APV). Top panels, Hemispheres segmented into ROIs. Bottom panels, 2D plots representing the ΔF/F as a function of time for the different ROIs. The numbering corresponds to the segmented area shown on top with lower values toward the anterior cortex (AC) and higher values toward the posterior cortex (PC). ***B***, Temporal evolution of calcium activity (ΔF/F) for the different anatomic regions before and after application of APV. Note that application of APV did not prevent the occurrence of large slow calcium events. ***C***, Scatter plots show the effect of APV on the amplitude and duration of the calcium events across the different anatomic regions. Note that blocking NMDA receptors primarily targeted calcium events with faster durations (i.e., <10 s). In the medial cortex (MC) from control slices, application of APV eliminated all activity. The ΔF/F amplitude at the PC in the presence of APV went below the cutoff threshold of 0.01 ΔF/F; thus, these data points are not shown. Additional details on the statistical analysis are found in Table 1 under the Figure 6 section.

**Figure 7.**
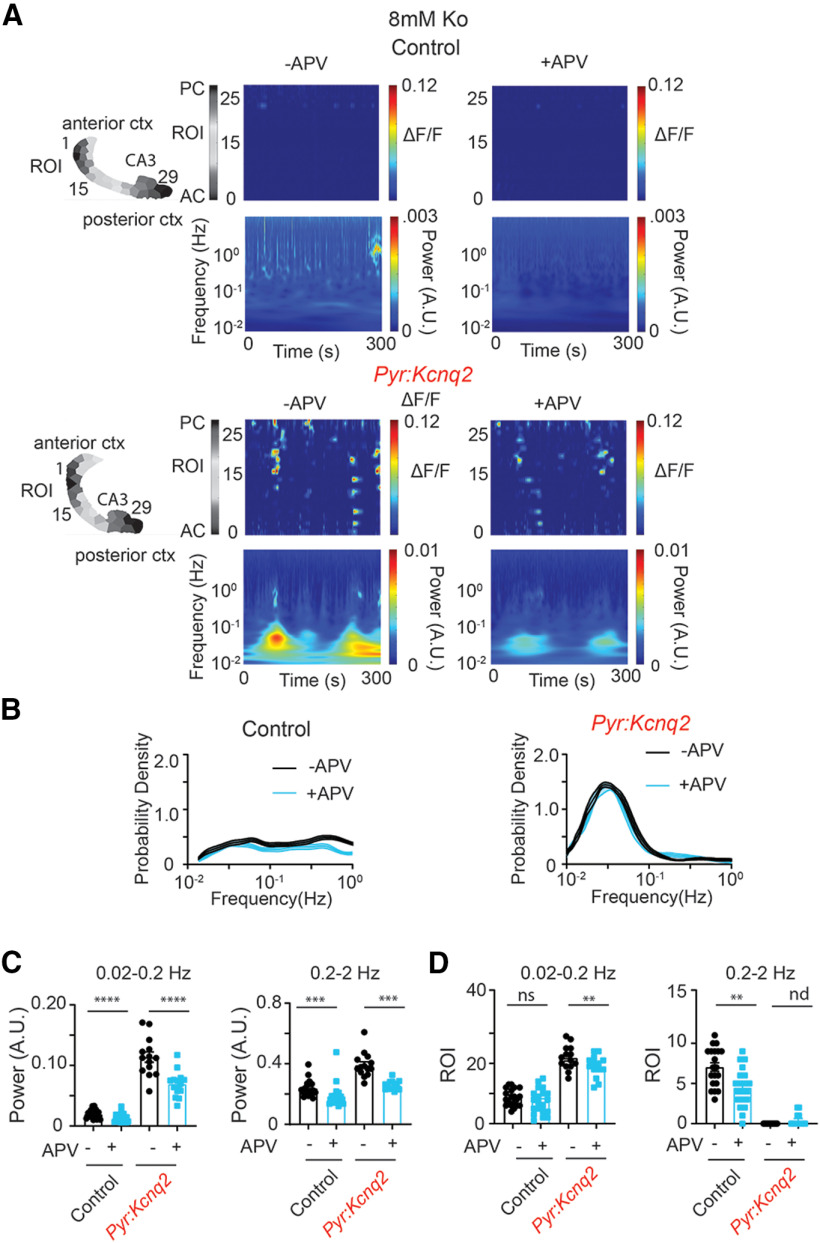
Blocking NMDA receptors does not prevent the emergence of slow oscillatory calcium activity in *Pyr:Kcnq2* slices. All recordings were in the presence of 8 mm Ko. ***A***, Representative examples of control and *Pyr:Kcnq2* 2D plots show calcium activity across ROIs and the corresponding wavelets. 2D plots and wavelets before and after application of 25 μm D-APV (APV) are shown. Note that APV reduced the power of the slow oscillatory activity in *Pyr:Kcnq2* slices. ***B***, Comparison of the probability density for the transient oscillation frequency (f) in the presence and absence of APV in control (*n* = 20) and *Pyr:Kcnq2* (*n* = 14) hemispheres. Note that APV did not change the likelihood of the emergence of a slow oscillatory activity at the 0.03- to 0.05-Hz frequency range. ***C***, Summary graphs show the effect of APV in control and *Pyr:Kcnq2* hemispheres on the power at the LF (0.02–0.2 Hz) and HF (0.2–2 Hz) domains. Note that APV reduced the power across all oscillatory frequencies, suggesting that NMDA receptors promote the calcium activity. ***D***, Summary graphs show the effect of APV on the number of ROIs in control and *Pyr:Kcnq2* slices, demonstrating sustained oscillations at frequency f*. Note that blocking NMDA receptors led to a decrease in the number of ROIs undergoing sustained oscillations in *Pyr:Kcnq2* hemispheres, consistent with our observed reduction in power shown in ***C***. Data are represented as mean ± SEM (**p < 0.01, ***p < 0.001, ****p < 0.0001). Additional details on the statistical analysis for panel ***C*** are found in Table 1 under the Figure 7 section. nd, not determined.

Consistent with this model, application of 25 μm D-2-amino-5-phosphonovalerate (D-APV) to our control slices reduced the amplitude of the calcium signals across all regions by ∼50–100% with the smallest effect at the CA3 region and the largest at the medial cortex (Fig. 6), thus suggesting that NMDA receptor activity is a key driver of network excitability in control slices. In contrast, inhibition of NMDA receptors had more complex effects in slices from *Pyr:Kcnq2* mice. First, blocking NMDA receptors reduced calcium activity by ∼30–50% in all forebrain regions (Fig. 6*B*,*C*). Second, blocking NMDA receptors prolonged the duration of calcium signals across all regions (Fig. 6*C*). This was because of the fact that NMDA receptor inhibition primarily targets large, faster events, causing the average signals to be mediated by longer lasting synchronous events (Fig. 6*C*). Accordingly, the time-frequency signal was significantly attenuated by application of APV (Fig. 7*A*) and resulted in a significant attenuation (i.e., 20–30%) of the power content both at LF and HF values (Fig. 7*C*). However, the application of APV did not alter substantially the sample distribution of transient oscillation frequencies (Fig. 7*B*) or the number of ROIs with significant sustained oscillations (Fig. 7*D*). Altogether, the behavior described in Figures 6, 7 is in contrast to the effect induced by blocking GABAA receptors and suggests that ongoing population network activity in *Kcnq2*-null excitatory neurons partially requires NMDA receptors. Furthermore, these results suggest that, in *Pyr:Kcnq2* slices, NMDA receptor inhibition primarily reduces the overall activity without necessarily suppressing large calcium events.

### Fast synaptic transmission does not prevent slow waves in slices from *Ppyr:Kcnq2* mice

**Figure 8.**
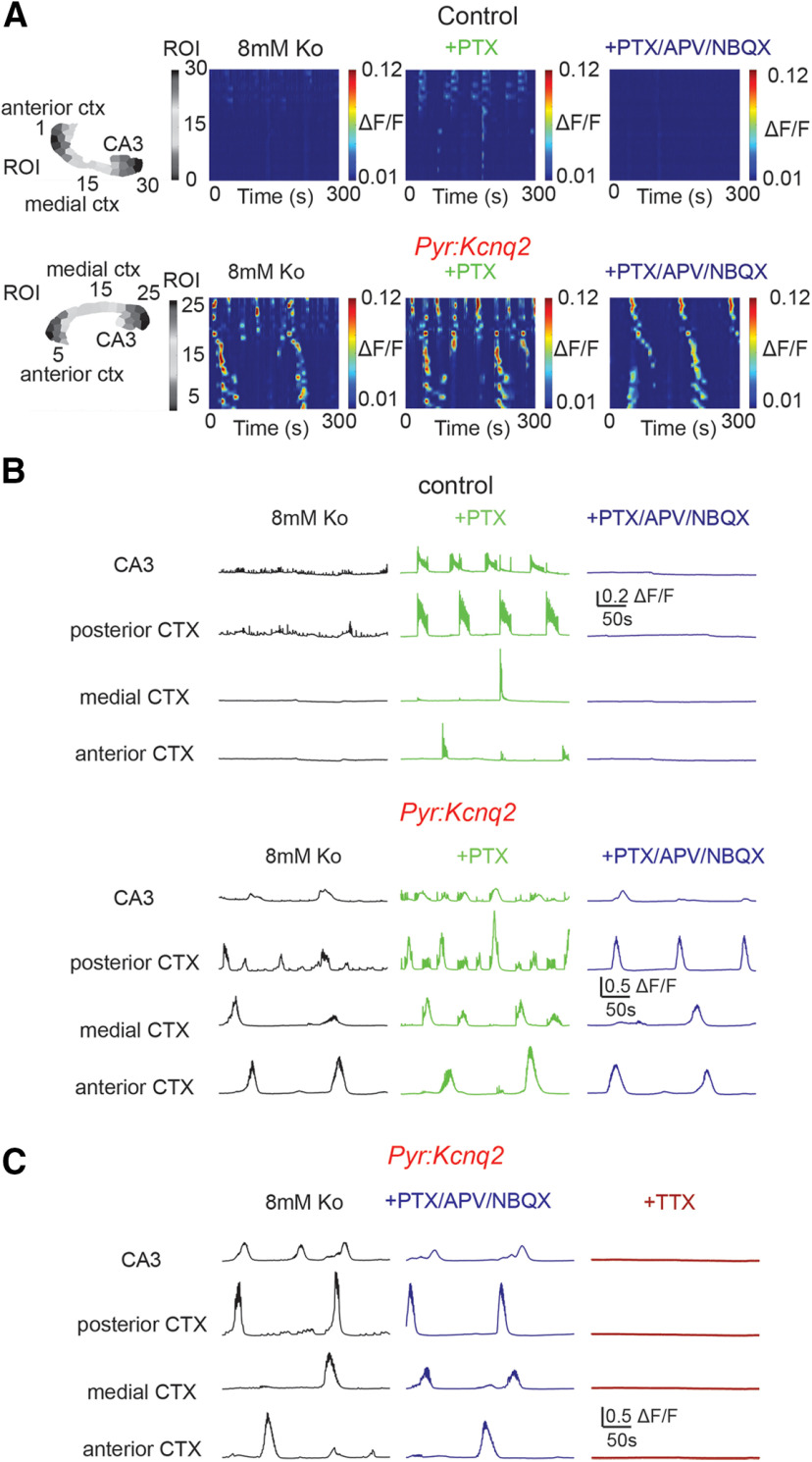
Calcium activity is maintained in the presence of fast excitatory transmission blockers, but eliminated by TTX in slices from *Pyr:Kcnq2* mice. All recordings were in the presence of 8 mm Ko. ***A***, top panels, Representative examples of control and *Pyr:Kcnq2* slices demonstrating calcium activity across all ROIs in the presence and absence of 50 μm PTX followed by 25 μm D-APV and 25 μm NBQX. The numbering corresponds to the segmented area shown on the left with lower values toward the anterior cortex (AC) and higher values toward the posterior cortex (PC). ***B***, Calcium activity (ΔF/F) across multiple ROIs representing different forebrain anatomic areas in the presence and absence of the GABAA receptor blocker PTX (middle panels) and glutamatergic transmission blockers APV and NBQX (right panels). Note that APV/NBQX primarily inhibited the smaller and faster calcium events that had emerged in the presence of PTX in *Pyr:Kcnq2* slices. Summary information regarding the amplitude, duration, and event frequency for multiple slices is found in Table 1 under the Figure 8 section. ***C***, 1 μm TTX (*n* = 6 slices) abolishes the calcium responses in slices preincubated with PTX/APV/NBQX.

To clarify whether the modulatory behavior reported above is restricted to NMDA receptors or rather extends to rapid synaptic transmission in general, we imaged control and *Pyr:Kcnq2* slices in the presence of the rapid synaptic transmission blockers 2,3-dioxo-6-nitro-7-sulfamoyl-benzo[*f*]quinoxaline (NBQX; AMPA receptor blocker), D-APV (NMDA receptor blocker), and PTX (GABAA receptor blocker). We found that blocking all fast synaptic transmission entirely abolished calcium activity in control slices (Fig. 8*A*,*B*), as reported previously (Sipila et al., 2005; Sheroziya et al., 2009). In contrast, in *Pyr:Kcnq2* slices activity was reduced across the forebrain, but still persisted significantly (Fig. 8*B*; Movie 3). In particular, we found a substantial reduction in pulsatile events of smaller amplitude and higher frequency, while large-amplitude calcium population events remained intact. Because of this, we observed an increase in the amplitude (posterior and medial cortex) and duration (CA3 region of the hippocampus, posterior and anterior cortex; Fig. 8*B*; see Table 1 for data summary). We note that the observed increases in duration and average amplitude were primarily because of the loss of small and short-lasting events; thus, the average duration was enriched by the long-lasting high-amplitude calcium events, rather than the emergence of new larger calcium events. We also did not find any effect of fast synaptic blockers on the propagation velocity of the calcium events (0.59 ± 0.11 mm/s, *n* = 20 hemispheres; 0.69 ± 0.13 mm/s, *n* = 18, *p* = 0.74 Mann–Whitney *U* test). In contrast to the experiments using fast synaptic blockers, calcium responses were eliminated on application of 1 μm tetrodotoxin (TTX; Fig. 8*C*), suggesting that voltage-gated sodium channel activity was required for the aberrant calcium responses in *Pyr:Kcnq2* slices.

**Figure 9.**
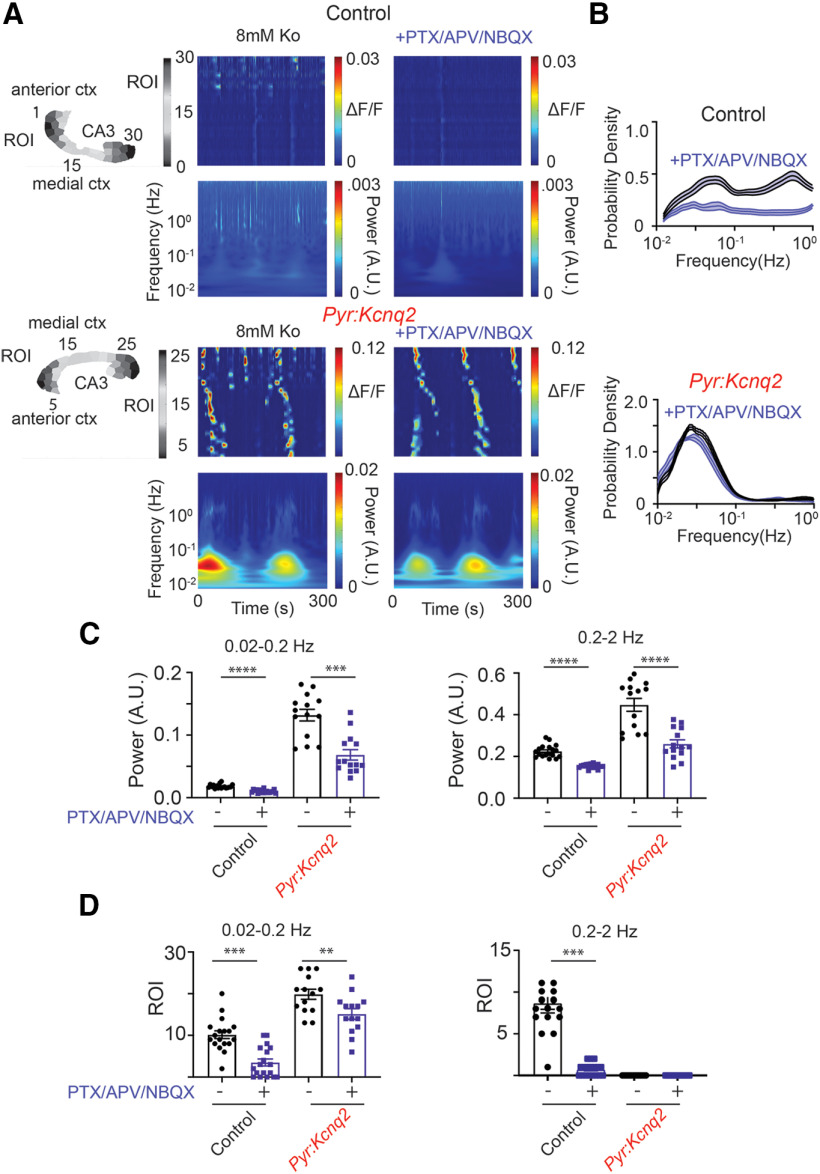
Differential effects of fast synaptic receptor blockers on the oscillation frequencies in control and *Pyr:Kcnq2* slices. All recordings were in the presence of 8 mm Ko. ***A***, Representative examples of control and *Pyr:Kcnq2* 2D plots and their corresponding wavelets in the presence and absence of 50 μm PTX, 25 μm D-APV, and 25 μm NBQX (PTX/APV/NBQX). Note that application of all synaptic blockers reduced the power at low frequencies, but did not prevent the emergence of slow oscillatory activity in the *Pyr:Kcnq2* hemisphere. ***B***, Comparison of the probability density of the frequency f of transient oscillations before and after application of PTX/APV/NBQX in control (*n* = 18) and *Pyr:Kcnq2* (*n* = 14) hemispheres. ***C***, Summary graphs show the effect of PTX/APV/NBQX on the power in control and *Pyr:Kcnq2* hemispheres for the LF (0.02–0.2 Hz) and HF (0.2–2 Hz) domains. ***D***, Summary graphs show the effect of PTX/APV/NBQX on the number of ROIs undergoing sustained oscillations in control and *Pyr:Kcnq2* hemispheres. Data are presented as mean ± SEM (**p < 0.01, ***p < 0.001, ****p < 0.0001). Additional details on the statistical analysis for panels ***C***, ***D*** are found in Table 1 under the Figure 9 section.

Similar to the data in Figure 8, we observed no significant changes in the time-frequency patterns in the *Pyr:Kcnq2* slices on suppression of fast synaptic transmission, i.e., the suppression failed to evoke substantial changes in the frequency distribution of transient oscillations across ROIs (Fig. 9*A*,*B*) or the number of ROIs that exhibited sustained oscillations (Fig. 9*D*). Instead, blocking fast synaptic transmission lowered the overall intensity of the transient oscillations (Fig. 9*A*) and resulted in lower spectral content in both the LF and HF bands (Fig. 9*C*). Finally, we did not observe these effects in the control slices; instead and in line with the expectations of blocking synaptic transmission, we found a robust suppression of transient oscillations across all ROIs (Fig. 9*B*) and a large reduction in the number of ROIs exhibiting sustained oscillations (Fig. 9*D*). Together, these data suggest that loss of KCNQ2 channels drives LF activity through a mechanism that is independent of fast synaptic transmission. Thus, in the absence of rapid synaptic transmission, slices from *Pyr:Kcnq2* mice can still generate large hypersynchronous events, likely because of sodium channel-driven depolarization of *Kcnq2*-null excitatory neurons and a large build-up and diffusion of potassium (Aiba and Noebels, 2021).

### Effects of *Kcnq3* deletion on network activity

Recent work has shown that KCNQ3 dysfunction leads to pharmaco-dependent epilepsy in patients lacking both functional *Kcnq3* copies (Lauritano et al., 2019), which is distinct from the pharmaco-resistant epilepsy caused by KCNQ2 dominant-negative variants (Cornet et al., 2018). Hence, we investigated whether loss of KCNQ3 activity leads to similar or different slow hypersynchronous events in the forebrain compared with loss of KCNQ2 activity. We thus repeated our experiments in *Kcnq3-*knock-out mice crossed with *Emx1^cre^:PC:Gcamp5*, as *Kcnq3*^−/−^ mice survive to adulthood.

**Figure 10.**
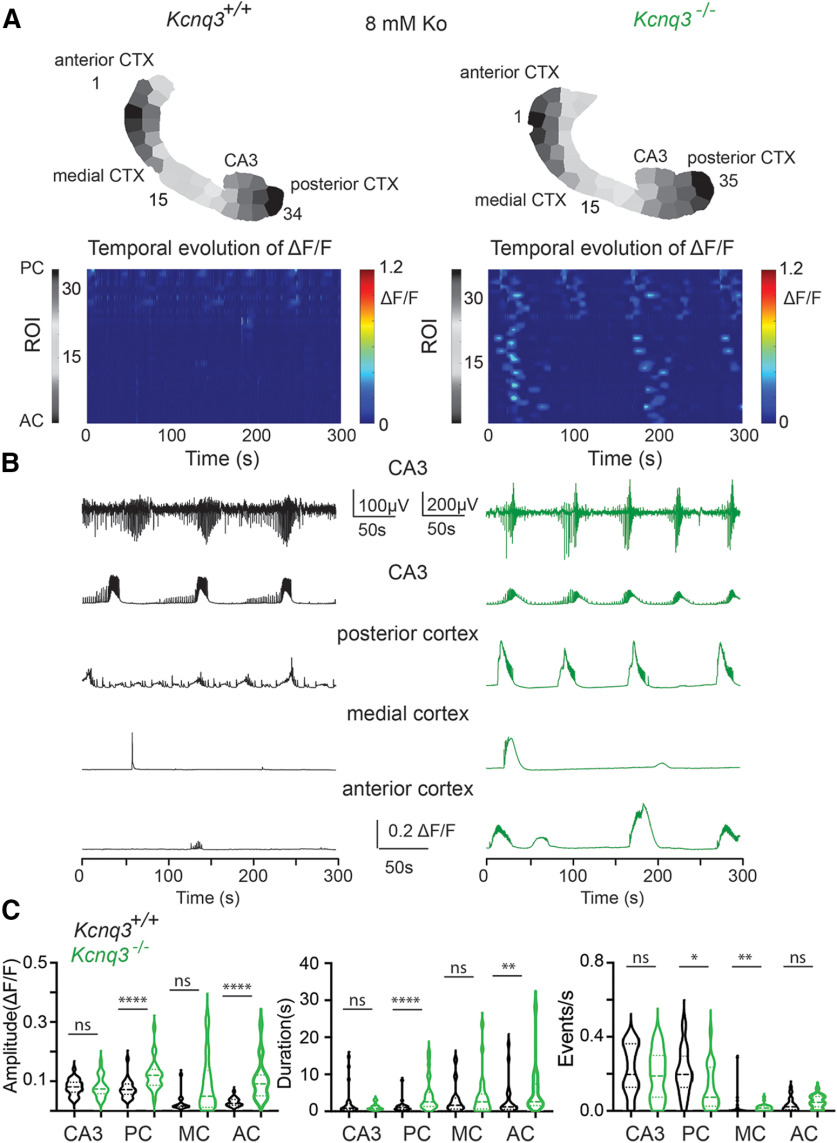
Ablation of *Kcnq3* leads to hyperexcitability across the forebrain in the presence of 8 mm Ko. ***A***, top panels, Two examples of acute slices from *Kcnq3^+/+^* and *Kcnq3*^−/−^ mice with one hemisphere segmented into ROIs. Below, 2D plots show the changes in the ΔF/F as a function of time for the different ROIs. Note that calcium activity is recorded across all regions in the *Kcnq3*-null slice. ***B***, Raw traces show calcium activity (ΔF/F) across the different anatomic areas. Top two panels, Temporal evolution of LFPs and ΔF/F recorded in parallel from the CA3 region of the hippocampus. ***C***, Violin plots show the effect of *Kcnq3* deletion on the ΔF/F amplitude, duration, and event frequency (events/s) for the different anatomic regions. The median and the interquartile ranges are also shown (*p < 0.05, **p < 0.01, ****p < 0.0001). Additional details on the statistical analysis for panel ***C*** are found in Table 1 under the Figure 10 section.

**Figure 11.**
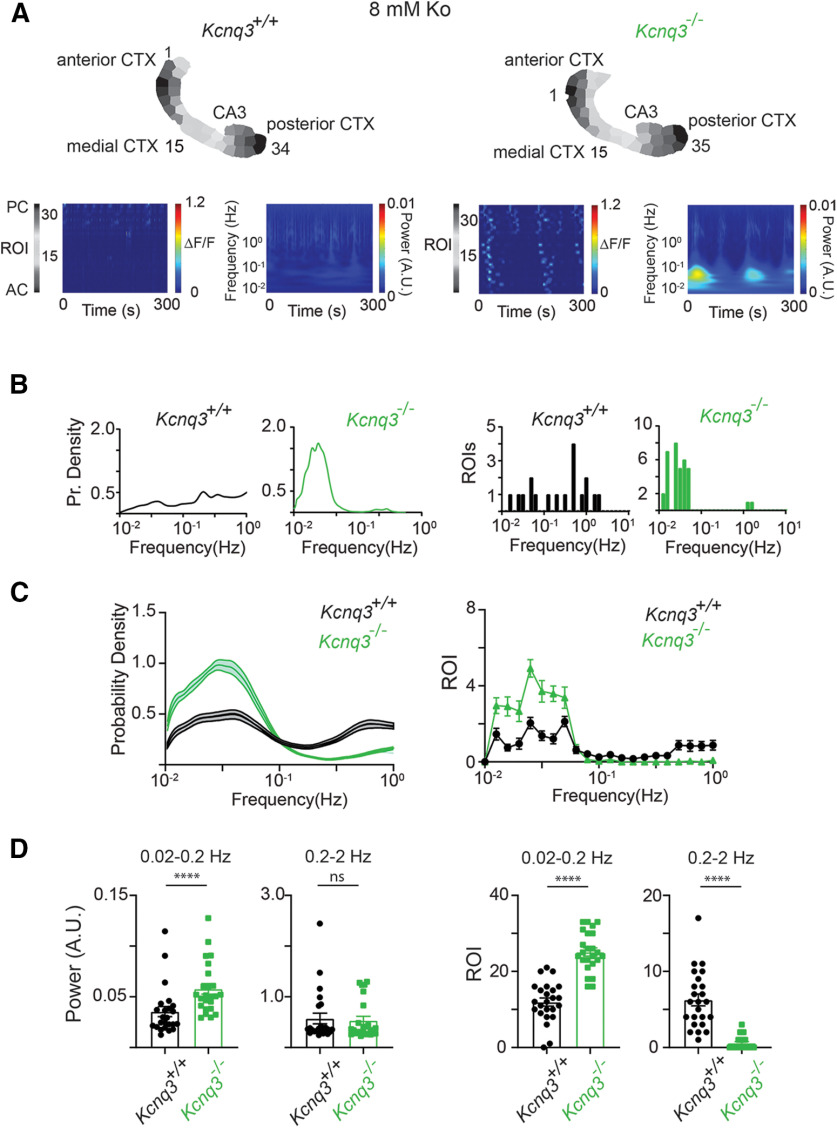
Loss of *Kcnq3* leads to LF calcium oscillations in multiple ROIs. All recordings were in the presence of 8 mm Ko. ***A***, Representative examples of *Kcnq3^+/+^*and *Kcnq3*^−/−^ ΔF/F 2D plots and wavelets. Top panels, ROIs used to generate the ΔF/F 2D plots and the wavelets. ***B***, Comparison of the probability density of the frequency f of transient oscillations and number of ROIs undergoing sustained oscillations for the examples shown in panel ***A***. ***C***, left, Comparison of the probability density of the frequency f of transient oscillations for *Kcnq3^+/+^*(*n* = 24) and *Kcnq3*^−/−^ (*n* = 24) across multiple hemispheres. Right, Comparison of ROIs undergoing sustained oscillations for *Kcnq3^+/+^*(*n* = 24) and *Kcnq3*^−/−^ (*n* = 24) hemispheres at different frequencies. ***D***, left panels, Summary graphs of the power at two frequency domains, 0.02–0.2 and 0.2–2 Hz. Right panels, Summary graphs of the ROIs exhibiting sustained oscillations at frequency $f^{*}$ in the range of 0.02–0.2 and 0.2–2 Hz. Data are represented as mean ± SEM (****p < 0.0001). Additional details on the statistical analysis for panel ***D*** are found in Table 1 under the Figure 11 section.

The activity in the *Kcnq3*-null slices was qualitatively similar to that in the slices without *Kcnq2*. Thus, ablation of *Kcnq3* also increases the network excitability across the forebrain and can lead to the emergence of large LF propagation calcium events (*Kcnq3*^−/−^ calcium propagation velocity = 0.73 ± 0.15 mm/s, *n* = 20 hemispheres; Fig. 10; Movie 4), thus suggesting a previously unappreciated role of KCNQ3 channels in the neonatal forebrain. However, we note several similarities and differences between the two mouse lines. (1) Unlike in *Pyr:Kcnq2* mice, ablation of *Kcnq3* did not increase the amplitude or the duration of calcium signals across all areas (Fig. 10*B*,*C*). For instance, we primarily found increases in the posterior cortex with less of an effect in the CA3 region of the hippocampus. (2) We observed an increase in the probability density, power, and ROIs primarily at the LF domain in *Kcnq3*^−/−^ slices. However, the overall increases were smaller than we observed in the *Pyr:Kcnq2* slices (compare Figs. 11 and 3). This smaller change in excitability because of *Kcnq3* ablation is consistent with the less severe phenotypes of *Kcnq3-*deficient than *Kcnq2*-deficient mice (Soh et al., 2014).

**Figure 12.**
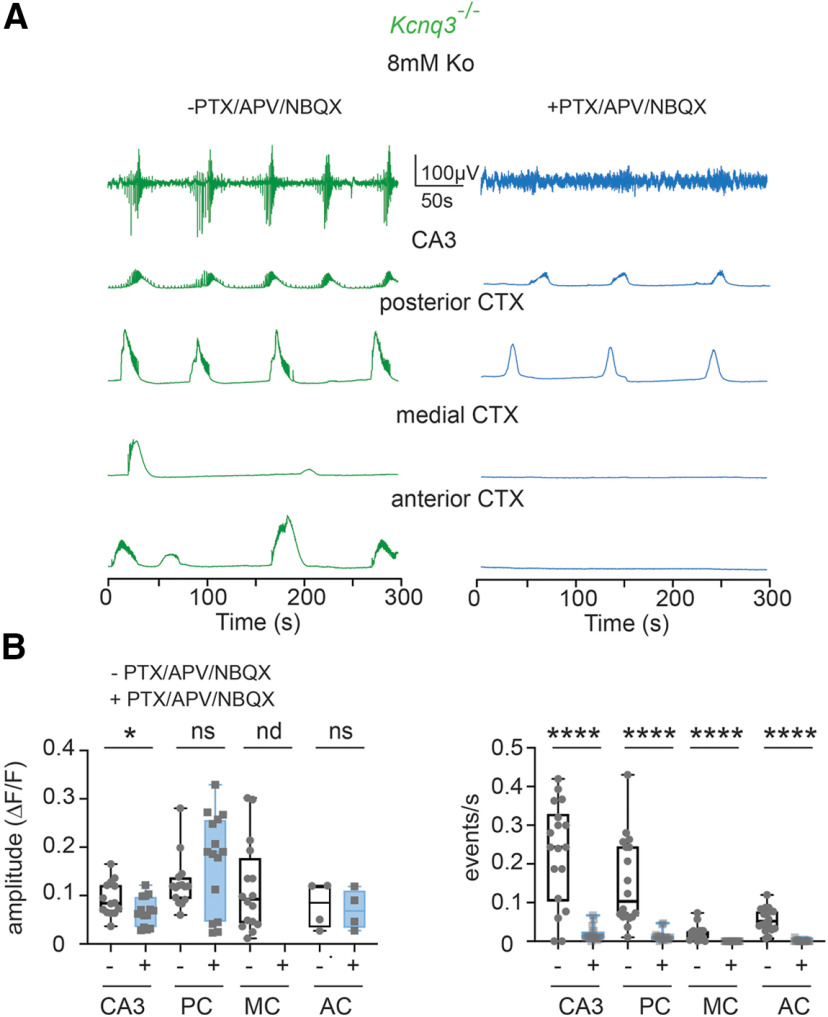
Synaptic blockers inhibit calcium activity in a region-specific manner in *Kcnq3*^−/−^ slices. All recordings were in the presence of 8 mm Ko. ***A***, top two panels, Recorded LFP and ΔF/F activity from the CA3 region of the hippocampus. Middle and bottom panels, Temporal evolution of the ΔF/F across different anatomic areas. Note that synaptic blockers (PTX/APV/NBQX) abolish the calcium activity only in the medial cortex (MC) and anterior cortex (AC). ***B***, Box plots show the effect of *Kcnq3*^−/−^ ablation on the ΔF/F amplitude, and event frequency for the different anatomic regions (*p < 0.05, ****p < 0.0001). Note that although application of synaptic blockers did not inhibit the amplitude of calcium events equally across the different regions, it did lead to a decrease to the number of calcium events across all regions. Additional details on the statistical analysis and number of replicates for panel ***B*** are found in Table 1 under the Figure 12 section.

**Figure 13.**
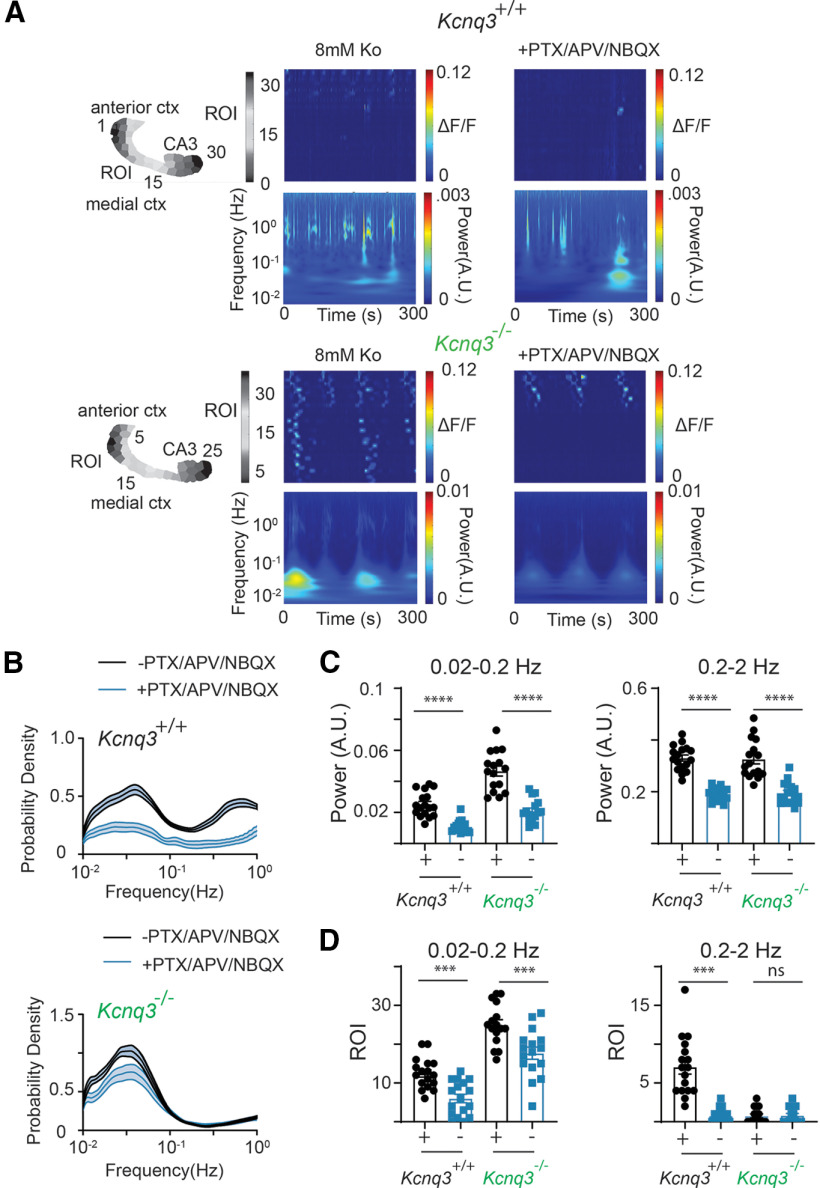
In *Kcnq3*^−/−^ slices synaptic blockers substantially reduce slow calcium oscillations in the neocortex. All recordings were in the presence of 8 mm Ko. ***A***, Representative examples of control and *Kcnq3*^−/−^ 2D plots along with their corresponding wavelets in the presence and absence of 50 μm PTX, 25 μm D-APV, and 25 μm NBQX (PTX/APV/NBQX). ***B***, Comparison of the probability density of the frequency f of transient oscillations before and after addition of PTX/APV/NBQX in *Kcnq3^+/+^* (*n* = 18) and *Kcnq3*^−/−^ (*n* = 18) hemispheres. Note that application of the synaptic blockers did not prevent the occurrence of the slow calcium oscillations in *Kcnq3*-null slices. ***C***, Summary graphs show the effect of PTX/APV/NBQX on the power for the LF (0.02–0.2 Hz) and HF (0.2–2 Hz) domains in *Kcnq3^+/+^*and *Kcnq3*^−/−^ hemispheres. Note that synaptic blockers reduce the power across the board, consistent with the large reduction of the activity in the neocortex. ***D***, Summary graphs show the effect of PTX/APV/NBQX on the number of ROIs in *Kcnq3^+/+^* and *Kcnq3*^−/−^ hemispheres that showed sustained oscillations at different frequencies. Data are presented as mean ± SEM (***p < 0.01, ****p < 0.001). Additional details on the statistical analysis for panels ***C***, ***D*** are found in Table 1 under the Figure 13 section.

Next, we tested whether the increase in LF calcium events in *Kcnq3-*knock-out mice is dependent on fast synaptic transmission. We found that the neocortical activity in *Kcnq3*-null mice was fully dependent on fast glutamatergic transmission, as co-application of NBQX and APV in the presence of PTX eliminated all ongoing activity at the neocortex (Fig. 12), this effect was not observed with *Pyr:Kcnq2* mice but was similar to what we found in control slices. Consequently, we found an overall decrease in the power and ROIs in slices from either *Kcnq3^+/+^* or *Kcnq3*^−/−^ slices (Fig. 13). Importantly, glutamatergic activity did not block the LF calcium events in the hippocampus similar to what we found in slices from *Pyr:Kcnq2* mice. This is best illustrated in Figure 13. Thus, KCNQ2 and KCNQ3 loss could lead to the generation of LF hypersynchronous events, but their dependence on fast synaptic transmission is specific to the forebrain region (see Discussion).

## Discussion

In this work, we determined the role of KCNQ2 and KCNQ3 channels in regulating the network activity of forebrain neonatal neuronal circuits. Although KCNQ2 and KCNQ3 channels are expressed in both GABAergic and glutamatergic neurons (Cooper et al., 2001), we primarily focused on forebrain excitatory cells as KCNQ2/3 channels regulate multiple facets of their excitability. The key findings of our study are that (1) KCNQ2 channel loss from pyramidal neurons can lead to multifocal spreading activity across all regions of the forebrain, (2) large LF calcium events modestly depend on fast synaptic transmission, and (3) unlike *Pyr:Kcnq2* mice, *Kcnq3*-null mice exhibit LF hypersynchronous activity driven by glutamatergic transmission in the neocortex but not the hippocampus. Our results provide new insights into the role of KCNQ2 and KCNQ3 channels in the neonatal brain and demonstrate that loss of KCNQ2 channels drives hyperexcitability in a manner distinct from KCNQ3-induced hypersynchrony in the neonatal neocortex.

### KCNQ2 channels in the developing forebrain

KCNQ2 channels are unique as they are expressed early in development, before birth (Kanaumi et al., 2008). For instance, studies examining KCNQ2 mRNA from human brain at different gestational periods have shown that KCNQ2 mRNA is expressed as early as 50 d postconception (http://development.psychencode.org/#). Consistent with this, KCNQ2 protein levels are highly expressed during the perinatal and early infantile period (Kanaumi et al., 2008). Thus, in retrospect it is not surprising that KCNQ2 pathogenic variants lead to range of pediatric epilepsy disorders from benign familial neonatal seizures to DEEs (Nappi et al., 2020).

The critical function of KCNQ2 channels early in development has also been supported in work with certain mouse models. For instance, loss-of-function *Kcnq2* homozygous mice die within 1 h following birth (Watanabe et al., 2000). Additionally, overexpression of dominant-negative isoforms of KCNQ2 channels during the first week of life, but not later, leads to lifelong seizures and premature death (Peters et al., 2005). Our work builds on these previous findings and makes several new contributions. Until now, most studies on the function of KCNQ2 channels in the developing cortex have focused on the hippocampus using pan-KCNQ channel blockers like XE991 and linopiridine (Okada et al., 2003; Qiu et al., 2007; Safiulina et al., 2008). However, such pharmacological reagents could not distinguish between KCNQ2 and KCNQ3, or parse through the contribution of different cell populations. By combining the use of *Kcnq2* conditional knock-out mice and mesoscale calcium imaging, we were able to define the function of KCNQ2 channels from excitatory neurons in the neonatal forebrain. Although activity under basal conditions was primarily confined to the hippocampus and posterior cortex, application of 8 mm extracellular potassium revealed a large hyperexcitable phenotype across the forebrain in slices from *Pyr:Kcnq2* animals. The activity was multifocal, that is multiple regions showed large population calcium events that on occasion would migrate across the neocortex. On many occasions, activity started at the entorhinal cortex, which then propagated to the neocortex or hippocampus; however, we also observed activity starting at the anterior cortical areas closer to the medial occipital cortex. Our data are consistent with an earlier imaging study from the neonatal brain that found that cortical waves typically originate from the entorhinal cortex (Namiki et al., 2013). We caution that our work was performed on acute slices that have many severed connections between regions; thus, in an intact brain, additional regions might also be hyperexcitable in the absence of KCNQ2 channels.

KCNQ2-containing channels have a critical role in controlling the excitability of the neonatal brain (Peters et al., 2005). Immature neurons have a depolarized membrane potential that would drive many voltage-gated potassium channels to either fast or slow inactivation, rendering them incapable of clamping down the membrane potential below the threshold to generate an action potential and preventing sodium channel inactivation (Telezhkin et al., 2018). KCNQ2 channels are slow-activating, non-inactivating potassium channels (Jentsch, 2000). Thus, KCNQ2 channels would remain open at resting membrane potentials of immature neurons (∼−30 mV to −50 mV depending on the age of the neuron) preventing unwanted excitation (Telezhkin et al., 2018). Additionally, the probability of KCNQ2 opening and voltage activation mid-point is modulated by the phospholipid PIP2 (Kim et al., 2016; Greene and Hoshi, 2017). This allows neurons to tailor KCNQ2 activity by changing membrane PIP2 levels. Thus, KCNQ2 channel gating properties and the subcellular localization, which early in development is both somatic and axonal, are suited for regulating the properties of immature neurons, before the full expression of additional voltage-gated and leak potassium channels.

### KCNQ2 versus KCNQ3 in neonatal excitability

Recent studies have shown that complete loss of KCNQ3 channel function can lead to pharmaco-dependent epilepsy and intellectual disability (Lauritano et al., 2019). Consistent with this, we found that loss of KCNQ3 activity leads to a hyperexcitable forebrain in the presence of an increased extracellular potassium concentration. Our data are consistent with earlier reports demonstrating mRNA expression of KCNQ3 from early developmental points and protein expression of KCNQ3 in human patients during pregestational time periods (Kanaumi et al., 2008). However, KCNQ3 loss had less robust effects than loss of KCNQ2 channels from the neocortex. This difference could be because of lower protein expression levels of KCNQ3 than KCNQ2 channels early in development or because of the presence of KCNQ2 channels, which could still function in the absence of KCNQ3 in neurons (Soh et al., 2014).

We also found that in *Kcnq3*-ablated mice, the aberrant neocortical activity was driven by synaptic activity, unlike the hypersynchrony because of KCNQ2 loss, further suggesting that loss of KCNQ2 or KCNQ3 lead to distinct effects in the developing brain. One possibility for the difference between the *Kcnq3*-deficient and *Kcnq2*-deficient neocortex might be that *Kcnq3* was removed from both interneurons and pyramidal neurons in contrast to *Kcnq2*. However, we do not think this could fully explain the difference between the mouse lines as the hyperexcitability in hippocampus was insensitive to synaptic transmission blockers in both *Kcnq2* and *Kcnq3* transgenic animals. Rather, the differences might reflect varying KCNQ2 and KCNQ3 protein expression level among the different regions early in development. Future studies are needed to decipher the role of KCNQ2 and KCNQ3 channels in the neonatal brain.

Movie 1. Representative imaging showing the forebrain calcium activity in control slices in the presence of 8 mM Ko. Note the activity in the hippocampal formation. Movie played at 5× speed. 10.1523/ENEURO.0024-21.2021.video.1

Movie 2. Representative imaging showing the forebrain calcium activity in *Pyr:Kcnq2* slices in the presence of 8 mm Ko. Note the migrating activity across the neocortex. Movie played at 5× speed. 10.1523/ENEURO.0024-21.2021.video.2

Movie 3. Representative imaging showing the forebrain calcium activity in the absence of *Kcnq2* in excitatory neurons and in the presence of synaptic blockers PTX, APV, and NBQX. Movie played at 5× speed. 10.1523/ENEURO.0024-21.2021.video.3

Movie 4. Representative imaging showing the forebrain calcium activity in the absence of *Kcnq3*. Movie played at 5× speed. 10.1523/ENEURO.0024-21.2021.video.4

### Possible model to explain our data

What could drive the slow hypersynchronous events? Based on knowledge from previous studies we propose the following working model. Under elevated excitability conditions such as in the presence of 8 mm extracellular potassium, a concentration that the brain could easily reach during robust activity or seizures, *Kcnq2*-null and (to a lesser extent) *Kcnq3*-null excitatory neurons would fire a barrage of action potentials or drive neurons to a prolonged afterdepolarization because of unabated persistent sodium channel activity. A prolonged somatic and axonal depolarization in the absence of synaptic blockers might further build up extracellular potassium because of the continual activation of voltage-gated potassium channels and the engagement of glutamate-gated NMDA and AMPA receptors. The large build-up of potassium would diffuse across neighboring regions, initiating waves of depolarization and network activity across the forebrain with its scope limited by ongoing GABAA receptor activity. Additional experiments are needed to test the different scenarios using additional transgenic mice.

In conclusion, our study fills an important knowledge gap by showing that loss of KCNQ2 and to a lesser extent KCNQ3 function from the neonatal forebrain can lead to a hypersynchrony in the neonatal brain that does not fully rely on the presence of fast glutamatergic transmission.

## Synthesis

Reviewing Editor: Douglas Bayliss, University of Virginia School of Medicine Decisions are customarily a result of the Reviewing Editor and the peer reviewers coming together and discussing their recommendations until a consensus is reached. When revisions are invited, a fact-based synthesis statement explaining their decision and outlining what is needed to prepare a revision will be listed below. The following reviewer(s) agreed to reveal their identity: Robert Foehring, Isamu Aiba. Note: If this manuscript was transferred from JNeurosci and a decision was made to accept the manuscript without peer review, a brief statement to this effect will instead be what is listed below.

This manuscript was assessed by two reviewers who found that the work was of high quality, with convincing results identifying effects of Kcnq2 and Kcnq3 deletion on neuronal network activity, with relevance for normal development and seizure pathologies. The principal concerns deal with the overall presentation, which should be simplified for clarity and ease of reading. A number of specific suggestions for revisions and corrections are provided with the submitted reviews (provided in full, below). In addition, a limitation regarding the relationship between recorded Ca2+ events and Na+ action potentials in neurons was noted. It was suggested in consultation session that this could be addressed by testing if the Ca2+ events in the slices are blocked by TTX, which would strengthen the paper; otherwise, this limitation should be explicitly acknowledged and discussed.

Reviewer #1

Advances the Field

The data clearly show an important role for KCNQ channels in preventing activity that spreads throughout cortex during development. This has implications for normal development as well as response to pathology such as seizures.

Multimedia Comments

The movies are essential.

Comments

The experimental design is rigorous and the data are high quality. The conclusions are well supported by the data. The figures and movies are clear and support the author's conclusions. One limitation of the work is that the proposed working model is dependent upon the spread of action potential-driven changes in extracellular K+ but the experiments do not clearly relate the recorded Ca2+ events to Na+ action potentials in neurons in the various cortical regions. Could this relate to the mismatch between Ca2+ events and changes to Local Field Potentials? What is the evidence that the Ca2+ responses are due to Na+ APs?

The manuscript is generally well-written but the sheer number of experiments and amount of data makes it a bit dense to read. In particular, The Figure Legends are difficult to follow due to the inclusion of copious summary data and statistics in addition to descriptions of the Figures. Perhaps this data would be better in several Tables (corresponding to each Figure)? The Figure Legends are also repetitive, which is perhaps unavoidable due to the experimental design, but this has led to some sloppiness in the headings (e.g., Figure 11 should be KCNQ3, not KCNQ2, it is not clear how the legend for Figure 12 relates to the data shown). Some summary of what the data show would be helpful in each Figure Legend.

Minor

1. Page 12, Statistics. Some explanation should be provided as to how data are displayed in box plots and especially, violin plots.

2. Page 15, lines 1-6. This sentence is a bit hard to follow. Is the point that the CRE is expressed early enough for you to be able to reduce KCNQ2 expression by P4 or is expression of KCNQ2 normally low before P4 (in which case, why should there be a difference from wild type at this age?).

3. Page 17, line 2. It is stated that there is “..widespread calcium activity spanning all cortical regions and layers” but in Movie 2-2, activity seems to be restricted to posterior and medial cortex but not anterior regions.

4. Page 26, Discussion line 1. Should this read “..we determined the role of KCNQ2 and KCNQ3 channels...”?

5. Page 28, line 1. This should read “.. was primarily confined to the hippocampal formation”.

6. Page 30, paragraph 1. Are there any differences in the cellular or subcellular expression of KCNQ2 vs. KCNQ3 in hippocampus or cortex?

7. Figure Legend 1. The heading says “...leads to hippocampal formation excitability” but the Figure also shows changes in posterior cortex. In (B), “graph” should be “graphs”. In (C), “..showing increasing firing” should read “showing increased firing...”.

8. Figure 2 legend. In lines 7-8 it should be stated that the temporal evolution of LFPs is also shown. Violin plots are used in this Figure but how they show aspects of the data is not explained.

9. Figure 3 Legend, lines 6 and 13. “(f*)” is undefined.

10. Figure 7 Legend. IN line 4, it should read “...oscillations before and after 25uM D-APV...”. In line 5, I believe “picrotoxin” should be “D-APV”.

11. Figure 11 Legend. I think the first sentence should read “Loss of KCNQ3 channels leads to an increase in slow frequency...”.

12. Figure 12 Legend. The first sentence is not clear to me. What is meant by “primarily inhibit neocortical activity..”? Do you mean fast synaptic blockers block neocortical activity but not hippocampal activity?

Reviewer #2

Advances the Field

KCNQ2 and KCNQ3 are linked to neonatal epileptic encephalopathy with various severities. However, their respective roles in the disease are not well understood, partly because of a lack of specific pharmacology. This study characterized the effects of Kcnq2 and Kcnq3 ablation on the neuronal network activity using wide-field Ca2+ imaging of acute neonatal brain slices prepared from Kcnq2 and Kcnq3 deficient mice.

The quality of the imaging study is high and the results are convincing. Compared with previous electrophysiology studies, the Ca2+ imaging approach provided a new anatomical vulnerability to these gene deficiencies. The results provided additional evidence for distinct roles of Kcnq2 and Kcnq3 in the regulation of neonatal neuronal activities.

Extended Data

The movies are excellent and should be published.

Comments

The experiments are appropriately conducted. I do not think any additional experiment is needed. On the other hand, there are some ambiguities, misuse of terminology, and typos in the text. I would like the authors to provide some additional information and clarify/correct the main text. I believe a proper revision will significantly improve this manuscript.

In the result section, ROI numbers and sizes were compared between WT and mutant slices and were similar, and the authors stated: “...indicate that no significant anatomical or functional difference between two groups...”. According to the manuscript, ROIs were determined by SLIC algorithm using fluorescence intensity. But it is not very clear whether ROIs are determined based on an image with Ca2+ activities or by using an image without any obvious Ca2+ activity (with inhibitor such as TTX)? Please explain more.

In Figure6, the figure legend “NMDA receptors driven hypersychrony in 8mM Ko is reduced in Pyr:Kcnq2 ex vivo slices.” seems somewhat misleading. Because Kcnq2 null slices had more activity, the result should be interpreted Kcnq2 deficient slices acquired NMDAR independent Ca2+ activities.

Figure11 legends, Loss of Kcnq3, not Kcnq2.

The movie is quite fascinating, especially the slowly migrating activities, but these events are not well described. Can authors provide an estimate of the velocities of these migrating activities?

The movie shows rather random spontaneous Ca2+ activities during each recording. Because of the short sampling period (300s), some of the results could be biased by sampling.

All experiments are conducted in the in vitro preparation, but preparation is not described in the title or abstract, until the very end of the introduction. This should be clarified at least in the abstract, otherwise, some readers may think this work was done in vivo.

Introduction Page 6 “...The KCNQ2 induced activity...” is not accurate, KCNQ2 did not induce Ca2+ activity. They are spontaneous neuronal activity detected in Kcnq2 null tissue. Please correct.

KCNQ2 and Kcnq2 are used in a mixed manner. In the mouse, Kcnq2 is correct. Please correct.

There are typos, in page8 Method, 4 mM NaCl. Also Page21, Ko not [K]o. Please carefully check for typos before the next submission.
